# A chromatin-associated protein required for inducing and limiting meiotic DNA double-strand break formation

**DOI:** 10.1093/nar/gky968

**Published:** 2018-10-24

**Authors:** Miao Tian, Josef Loidl

**Affiliations:** Department of Chromosome Biology, Max F. Perutz Laboratories, University of Vienna, 1030 Vienna, Austria

## Abstract

Programmed DNA double-strand breaks (DSBs) are required for meiotic recombination, but the number is strictly controlled because they are potentially harmful. Here we report a novel protein, Pars11, which is required for Spo11-dependent DSB formation in the protist *Tetrahymena*. Pars11 localizes to chromatin early in meiotic prophase in a Spo11-independent manner and is removed before the end of prophase. Pars11 removal depends on DSB formation and ATR-dependent phosphorylation. In the absence of the DNA damage sensor kinase ATR, Pars11 is retained on chromatin and excess DSBs are generated. Similar levels of Pars11 persistence and DSB overproduction occur in a non-phosphorylatable *pars11* mutant. We conclude that Pars11 supports DSB formation by Spo11 until enough DSBs are formed; thereafter, DSB production stops in response to ATR-dependent degradation of Pars11 or its removal from chromatin. A similar DSB control mechanism involving a Rec114-Tel1/ATM-dependent negative feedback loop regulates DSB formation in budding yeast. However, there is no detectable sequence homology between Pars11 and Rec114, and DSB numbers are more tightly controlled by Pars11 than by Rec114. The discovery of this mechanism for DSB regulation in the evolutionarily distant protist and fungal lineages suggests that it is conserved across eukaryotes.

## INTRODUCTION

During meiosis, numerous DNA double-strand breaks (DSBs) are generated to ensure proper homology searching and homologous pairing [see (1)]. A subset of DSBs is converted into inter-homolog crossovers (COs), which contribute to parental gene shuffling in the gametes and the genetic diversity of offspring. The central factor of DSB formation is the meiosis-specific transesterase Spo11, which induces DSBs in a topoisomerase-like reaction [see (1)]. Both evolutionary conserved and group-specific co-factors have been identified that aid Spo11 in targeting and cleaving DNA [(2–9); see (10,11)]. This auxiliary complex is best understood in budding yeast, where it consists of several subgroups. One of these, RMM (consisting of Rec114, Mer2 and Mei4), resides on chromatin and is required for Spo11 recruitment to the meiotic chromosome axis (12–14).

Shortly after their formation, meiotic DSBs are occupied by the MRN/MRX-complex, which recruits and activates the DNA damage sensor kinase ATM, a phosphatidylinositol 3-kinase-related kinase (PIKK) [see (15,16)]. In turn, ATM promotes the removal of covalently bound Spo11 and 5′ strand resection by ATM-dependent phosphorylation of Mre11, which is part of the MRN/MRX complex (17). Subsequently, 3′ single-stranded overhangs are coated with RPA, eliciting a DNA damage signal by another PIKK, ATR [see (18)]. Activated ATM also phosphorylates numerous other proteins that function in meiotic chromosome pairing and DSB repair (19).

To ensure correct DSB-dependent chromosome pairing and obligatory CO formation, a marked excess of DSBs is generated, with the majority of DSBs being converted into non-CO recombination outcomes [see (20)]. The factors that induce DSBs are sufficiently abundant to generate even more DSBs, but the capacity of the repair machinery might be overwhelmed by too many DSBs. Therefore, mechanisms to limit the number of DSBs exist in different model organisms [see (21)].

To study whether and how such mechanisms operate in an evolutionarily distant organism, we turned to the protist *Tetrahymena thermophila. Tetrahymena* is a unicellular organism that can propagate both vegetatively and by sexual reproduction. It has two functionally distinct nuclei (22). One is the transcriptionally silent germline nucleus (micronucleus) with 2n = 10 chromosomes. It divides mitotically during vegetative growth, and undergoes meiosis and is passed on to the offspring during sexual reproduction. The other is the transcriptionally active polyploid somatic nucleus (macronucleus). It divides by amitotic splitting and becomes degraded during sexual reproduction.

**Figure 1.**
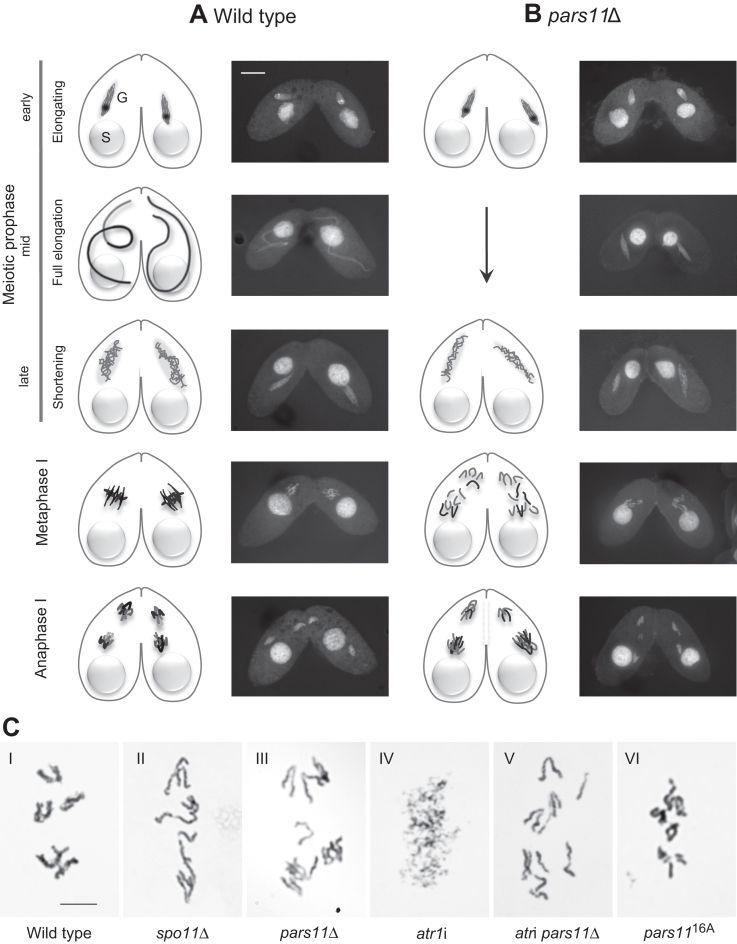
The meiotic process in WT and mutant *Tetrahymena*. (**A**) Meiosis in the WT. Starved cells of complementary mating types associate and their diploid germline nuclei (G) undergo roughly synchronous meioses. The polyploid somatic nuclei (S) remain unchanged. Early meiotic prophase nuclei become spindle shaped. As soon as meiotic DSBs are induced, they trigger the further elongation of nuclei to about twice the length of the cell. Homologous pairing takes place in fully elongated nuclei. After the pairing stage, the nucleus shortens; DSBs are repaired only during nuclear shortening (26). At the end of meiotic prophase, five condensed bivalents become visible. Bivalents assemble in an equatorial plate at metaphase I and homologous chromosomes separate at anaphase I. (**B**) Meiosis in *pars11*Δ. The fully elongated prophase stage is skipped. Instead of bivalents, univalents are formed; they do not assemble in a metaphase plate and segregate randomly during anaphase I. (**C**) Giemsa-stained diakinesis and corresponding stages. (C-I) Five bivalents in the WT. (C-II) Univalents in *spo11*Δ. (C-III) Univalents in *pars11*Δ. (C-IV) Mass of fragmented chromatin in *atr1*i. (C-V) Intact univalents in the *atr1*i *pars11*Δ double mutant. (C-VI) Largely intact chromosomes/bivalents in the *pars11*^16A^ mutant. Bar in A: 10 μm, bar in C: 5 μm.

Upon starvation, two cells of complementary mating types can mate and initiate synchronous meioses [see (23)]. DSBs are formed by Spo11 early in meiotic prophase. These DSBs are sensed by ATR [ATM is notably lacking in *Tetrahymena* (24,25)], and trigger the elongation of germline nuclei to about twice the length of the cell (Figure 1A). Within the elongated nucleus, chromosomes are arranged in a stretched bouquet-like manner, with centromeres and telomeres attached to opposing ends. This arrangement promotes the juxtapositioning of homologous regions, thereby facilitating homologous pairing and CO without the help of a synaptonemal complex (26). Following this unusual pairing stage, the nuclei shorten and DSBs are repaired with the help of Rad51 and Dmc1 [see (27)]. Condensed bivalents become discernible at the diplotene/diakinesis stage (Figure 1C–I), which is followed by closed first and second meiotic divisions (Figure 1A).

Here, we characterize a novel gene, *PARS11* (*Partner of SPO11*), which is required for both the DSB formation and ATR-dependent control of DSB number.

## MATERIALS AND METHODS

### Strains, cell growth and induction of meiosis

*Tetrahymena thermophila* wild-type (WT) strains B2086 (mating type II) and Cu428 (mating type VII) were obtained from the Tetrahymena Stock Center at Cornell University. The *spo11* and *com1* knockout lines were described previously (28,29). Cells were grown in Neff medium at 30°C according to standard methodology [see (30)], and were made competent for sexual reproduction by starvation in 10 mM Tris–Cl (pH7.4) for at least 16 h. Cell mating and meiosis were induced by mixing starved cultures of different mating types.

To induce artificial DNA damage, cells were pretreated ∼2 h after induction of meiosis with 100 μg/ml cisplatin, γ-irradiation (5000 rad from a ^60^Co source) or UV-C irradiation (354 nm, 50 J/m^2^). ATR was chemically inhibited with 10 mM caffeine applied ∼2 h after induction of meiosis (25). To detect recombination-related DNA synthesis, bromodeoxyuridine (BrdU) (final concentration: 2 × 10^−4^ M) was added 2.5 h after induction of meiosis.

### Construction of *pars11* knockout strains

A plasmid cassette carrying ∼650 bp sequences flanking the *PARS11* ORF and a selectable *CHX* (cycloheximide resistance) marker was constructed by Gibson assembly using the primers listed in Supplementary Table S1. The knockout cassette (Supplementary Figure S1A) was introduced into *Tetrahymena* cells by biolistic transformation to replace the WT locus by homologous recombination (31). Transformants were selected by growth in medium containing increasing concentrations (from 15 to 240 μg/ml) of cycloheximide and 4.5 μg/ml Cadmium chloride (32). To elicit the knockout phenotype in meiosis, both mating partners had to be transformed, because mating cells can rescue their partner’s defects by exchanging gene products (33). Complete knockout was confirmed using quantitative polymerase chain reaction (qPCR) and reverse transcription PCR (Supplementary Figure S2).

### ATR depletion by RNA interference

To create an *atr1*RNAi construct, a fragment of the *ATR1* ORF (from 1295 to 1952 bp) was amplified from genomic DNA using the PCR primers listed in Supplementary Table S1. Two copies of this fragment were inserted end-to-end behind a Cd^2+^-inducible *MTT1* metallothionein promoter into the pAkRNAi-Neo5 plasmid (see Supplementary Figure S1B) (34,35). The vector also contained a codon-optimized *NEO5* (neomycin/paromomycin resistance) cassette under the constitutively expressed *HHF1* (*Histone H Four 1*) promoter (36). The construct was introduced into starved cells by biolistic transformation. Transformants were selected by growth in medium containing increasing concentrations of paromomycin. For efficient ATR depletion in meiotic cells, hairpin dsRNA expression was induced by the addition of 0.075 μg/ml CdCl_2_ during pre-meiotic starvation. High Knockdown efficiency was confirmed by the strong reduction in fully elongated nuclei [ATR is required to induce elongation (25)]: after cadmium-induced cells were mated to uninduced cells, a fully elongated nucleus was present at 3.5 h after induction of meiosis in both partners of only two out of 100 mating pairs.

### Pars11 tagging and amino acid substitution

For C-terminal terminal hemagglutinin (HA) epitope tagging, the coding sequence of the *PARS11* gene up to the TGA stop codon was amplified from genomic DNA with a forward primer containing an adaptor sequence for Gibson assembly and a reverse primer containing HA-coding sequences. A ∼500 bp 3′-UTR fragment downstream of the TGA was amplified with a forward primer containing the HA-coding sequence and a reverse primer containing an adaptor sequence for Gibson assembly. Another downstream ∼600 bp 3′-UTR fragment was amplified with primers containing adaptor sequences. A *NEO4* (paromomycin resistance) cassette was inserted between the two UTR sequences, and all were fused and cloned into the Not I site of the pBluescript SK(-) vector (Stratagene, La Jolla, CA, USA) by Gibson assembly (Supplementary Figure S1C). The construct was then linearized by Not I digestion and transformed into the endogenous locus by particle bombardment. Transformants were selected by growth in medium containing increasing concentrations of paromomycin and complete replacement of WT Pars11 was confirmed by qPCR. DSB and bivalent formation was normal (data not shown).

For the mutation of the 16 N-terminal S/T-Q sites of Pars11, Ser and Thr codons were replaced by a 603-bp synthesized tract carrying the respective Ala codons. The modified sequence was fused with the *PARS11* 5′-UTR sequence, the C-terminal ORF sequence, the 3′-UTR sequence and a *NEO4* or *CHX* cassette and cloned into the NotI site of pBluescript SK(-) by Gibson assembly. The *NEO4* construct was then linearized by NotI digestion and transformed into the *pars11*Δ mutants at the original locus (Supplementary Figure S1D). The *CHX* construct was digested by NotI and transformed into *com1*Δ mutants (29). Transformants were selected by growth on medium with paromomycin or cycloheximide.

### PFGE and Southern hybridization

For pulsed-field electrophoresis (PFGE), genomic DNA from efficiently mating cultures was isolated and embedded in low-melt agarose plugs. The PFGE run was performed in 1% agarose with 0.5 × TBE buffer at 6 V/cm and 14°C for 14 h with 60-s pulses, 10 h with 90-s pulses and 1 h with 120-s pulses. The vast majority of cellular DNA resides in minichromosomes of the somatic nucleus; this enters the gel and masks the DSB-dependent fragments of the germline nucleus. Therefore, Southern-blotting was done to hybridize the gel to a radiolabeled germline-specific probe [see (29)]. Gels were stained with ethidium bromide to ensure equal loading of meiotic DNA samples from different genotypes, and only experiments with ≥80% of mating cells were evaluated. Line profile analyses of gels were carried out using the Line Profile tool in AutoQuant X3 software.

### Western blotting

To detect protein by western blotting, 1.5 ml cell samples were harvested at different timepoints after induction of meiosis. Proteins were precipitated with 10% trichloroacetic acid (TCA) on ice for 10 min and pelleted by centrifugation at 2500 *g* for 5 min. Remaining TCA was removed from the pellet by washing with ice-cold 1 × phosphate-buffered saline (PBS) buffer. The pellet was resuspended in ice-cold 1 × PBS by vigorous vortexing and then one volume of 5 × sodium dodecyl sulphate loading buffer was added to four volumes of resuspended pellet and boiled for 10 min. Samples of equal volumes were separated by sodium dodecyl sulphate-polyacrylamide gel electrophoresis (SDS-PAGE) and analyzed by western blotting using the indicated antibodies. HA-tagged protein was detected with mouse monoclonal anti-HA antibody (1:1000 dilution; clone HA-7, Sigma-Aldrich, St Louis, MO, USA), and α-tubulin was detected with mouse monoclonal anti-α-tubulin antibody (1:5000 dilution; Clone DM1A, Thermo Fisher Scientific, Waltham, MA, USA). Primary antibodies were detected using HRP-conjugated anti-mouse antibody (1:5000 dilution).

For phosphatase treatment and band shift assays, a crude protein extract from 1.5 ml *Tetrahymena* cells was washed twice with ice-cold 1 × PBS buffer containing cOmplete proteinase inhibitor (Roche Diagnostics, Indianapolis, IN, USA), resuspended in 60 μl 1 × CutSmart buffer (NEB, Ipswich, MA, USA) and treated with calf intestinal alkaline phosphatase (NEB) at 37°C for 1 h. A 20 μl sample was then resuspended in 1 × SDS-PAGE loading buffer and boiled for 10 min. Protein extracts were separated on a 7.5% SDS-PAGE gel to resolve phosphorylated and non-phosphorylated Pars11-HA species, which were detected by western blotting with a mouse monoclonal anti-HA antibody.

### Cytological techniques

#### Conventional DAPI staining and immunostaining

Aliquots (5 ml) of cell suspension were transferred to a centrifuge tube and 250 μl 10% Triton X-100 and 500 μl of 37% formaldehyde were added. After fixation at room temperature for 30 min, the cell suspension was centrifuged for 2 min at 400 *g* and the pellet was resuspended in 500 μl of a solution (4% paraformaldehyde plus 3.4% sucrose). A drop of this mixture was spread onto a slide and air-dried. To detect incorporated BrdU, DNA on the slides was denatured by incubation with 70% formamide for 2 min at 65°C. Slides were rinsed in 1 × PBS (twice for 5 min) and then in 1 × PBS containing 0.05% Triton X-100 (5 min). Anti-BrdU-antibody (1:40 dilution; rat anti-BrdU, Abcam, Cambridge, UK), anti-HA antibody (1:200 dilution; rabbit polyclonal) or anti-Dmc1/Rad51-antibody (1:50 dilution; mouse, clone 51RAD01, NeoMarkers, Fremont, CA, USA) was applied under a coverslip. After washing as above, the appropriate fluorescence-labeled secondary antibodies were applied. Slides were mounted under a coverslip in Vectashield anti-fading agent (Vector Laboratories, Burlingame, CA, USA) supplemented with 0.5 μg/ml DAPI for inspection by fluorescence microscopy. For details see (26).

#### Preparation of high-detergent spreads for selective immunostaining of chromatin-associated proteins

Aliquots (5 ml) of conjugating cell suspension were transferred to a centrifuge tube and 500 μl of a mixture (450 μl 10% Triton plus 50 μl 37% formaldehyde) was added. After 25–30 min on ice, another 450 μl 37% formaldehyde was added. After 5 min, the cells were centrifuged, and the pellet was resuspended in 500 μl of a solution (4% paraformaldehyde and 3.4% sucrose). A drop of this mixture was spread onto a slide and air-dried. Primary and fluorescence-labeled secondary antibodies were applied as described above.

#### Cna1 and γ-H2A.X immunostaining

Aliquots (5 ml) of cell suspension were transferred to a centrifuge tube and 20 μl partial Schaudinn’s fixative (2:1 ratio of saturated HgCl_2_,: absolute ethanol) were added. After 5 min, cells were washed twice with methanol and then resuspended in 50 μl methanol; drops of this suspension were applied to a slide and air-dried. Immunostaining of Cna1, a CENP-A homolog, used a rabbit polyclonal antiserum (1:200 dilution) [a gift from Harmit Malik, see (26,37)] and γ-H2A.X immunostaining used a mouse monoclonal antibody (1:200 dilution; clone 2F3, BioLegend, San Diego, CA, USA). Both were detected with a fluorescence-labeled secondary antibody.

#### Giemsa staining

Giemsa staining of Schaudinn-fixed cell preparations provides superior resolution of metaphase I chromosomes. Aliquots (5 ml) of cell suspension were transferred to a centrifuge tube and centrifuged (400 *g* for 3 min). The pellet was resuspended in 500 μl Schaudinn’s fixative (330 μl saturated HgCl_2_, 165 μl absolute ethanol, 5 μl acetic acid). After 1 h, cells were washed and resuspended in 1 ml 70% ethanol. Pelleted cells from 100 μl of this suspension were resuspended in 300 μl methanol/acetic acid mixture (3:1 ratio) and then dropped onto a slide and air-dried. Cells were hydrated and incubated with 100 μl 5 N HCl under a coverslip for 2 min, washed in distilled water, and air dried. Slides were incubated in 4% Giemsa solution in 1 × PBS for 10 min, washed under running water, and air dried before mounting with Euparal and inspecting by light microscopy.

## RESULTS

### *pars11*Δ cells undergo abnormal meiosis

We performed a systematic deletion screen of meiotically expressed genes in *Tetrahymena* using the rapid co-deletion procedure by (38). It identified a mutant of a novel gene, *PARS11* (transcribed from ORF TTHERM_00133730 in the Tetrahymena Genome Database—http://ciliate.org/), with defects in homologous pairing and recombination. Subsequently, conventional knockout strains were produced for a closer analysis of the knockout phenotype.

The most notable anomaly in *pars11*Δ meiosis is the failure of prophase nuclei to fully elongate. Instead, nuclei gradually transit from early prophase (corresponding to the pre-elongation stage of the WT) to late prophase (corresponding to the WT post-elongation stage) (Figure 1B). The early and late prophase substages could be discriminated by smooth and filamentous chromatin structure, respectively, which is probably due to increasing chromatin condensation. In diakinesis 10 univalents appear (Figure 1C-III). They do not aggregate in a metaphase plate but immediately enter anaphase I by moving from dispersed nuclear location toward the respective nearest pole, which, in most cases, leads to unequal separation (Figure 1B). This closely resembles the behavior of the *spo11*Δ mutant, in which DSBs are not formed (Figure 1C-II and (28). Defective meiosis in *pars11*Δ was also indicated by the failure of 95.6% (*n* = 500) of progeny cells to replace the old somatic nucleus with a functional new somatic nucleus 24 h after initiation of meiosis.

**Figure 2.**
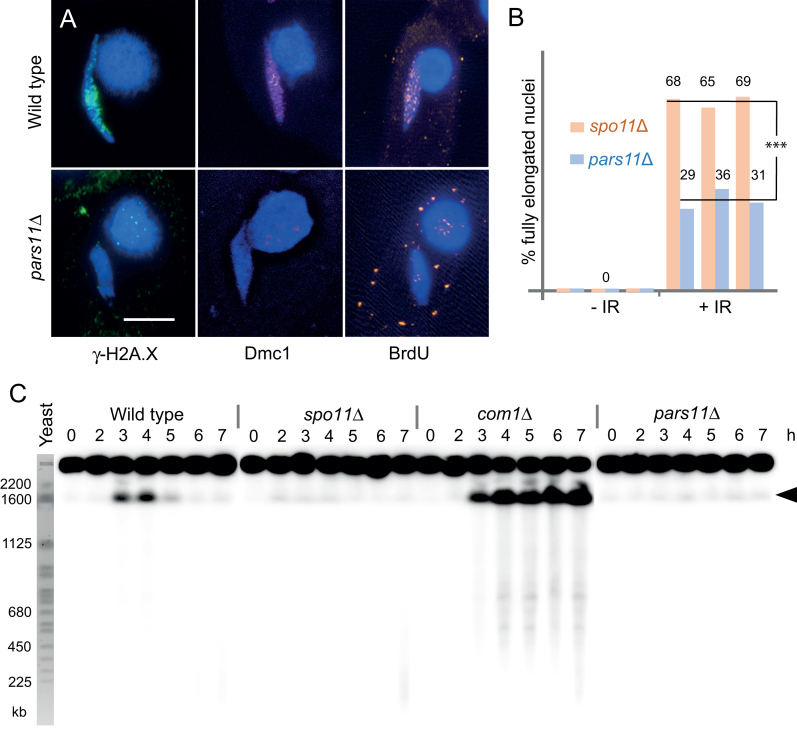
DSBs are not formed in *pars11*Δ. (**A**) Markers of DSB formation, sensing and repair. Histone variant H2A.X is phosphorylated (γ-H2A.X), Dmc1 localizes to chromatin and BrdU is incorporated into meiotic nuclei in the WT but not *pars11*Δ. Bar: 5 μm. (**B**) Nuclear elongation is partially but differently (***P < 0.001, *t*-test) restored in *spo11*Δ and *pars11*Δ by γ-radiation (IR)-induced DNA damage. Three biological repeats with 100 cells each for *spo11*Δ and *pars11*Δ were evaluated. (**C**) DSBs are visualized by PFGE. DSB-dependent DNA fragmentation is transient in the WT and permanent in the *com1*Δ mutant. Most DSB-dependent DNA fragments migrate as a single band (arrowhead). Only slight chromosome fragmentation (possibly due to DNA damage during sample preparation) is seen in *spo11*Δ and *pars11*Δ. Budding yeast chromosomes are shown as size markers. h: hours after induction of meiosis.

In the WT, besides elongation of the meiotic prophase nucleus, histone H2A.X phosphorylation (γ-H2A.X) (28,39) and chromatin localization of the recombination protein Dmc1 (40) indicate the formation and processing of DSBs, and BrdU incorporation indicates recombinational repair of DSBs (26). None of these markers of normal recombination were observed in *pars11*Δ meiosis (Figure 2A), suggesting that DSBs are either not formed or not sensed, and hence not repaired by a canonical recombinational repair mechanism. Notably, in contrast to a *spo11*Δ mutant, in which nuclear elongation can be restored by a variety of physical or chemical DNA damaging agents (25), elongation was only weakly induced by γ-irradiation (Figure 2B). The weak response to artificial DNA lesions suggests that the DNA damage response is attenuated in *pars11*Δ.

### DSB formation requires Pars11

To determine whether the *pars11*Δ mutant produces DSBs, a DNA fragmentation assay was performed (Figure 2C). In this assay, the chromosome fragments generated by DSBs are separated by PFGE whereas intact germline chromosomes [25.5–36.3 Mb; (41)] do not enter the gel. Since chromosome fragments from the germline nucleus are masked by the large excess of DNA from the somatic nucleus, they were marked by Southern hybridization using a specific probe for germline DNA (29). In *Tetrahymena*, meiotic DSBs generate DNA fragments, most of which are between 1000 and 4600 kb (34). Under our standard PFGE conditions, fragments larger than ∼2000 kb are not resolved and run as a distinct band, whereas smaller fragments produce a weak smear. In the WT these fragments are transiently formed at ∼3–5 h after induction of meiosis and indicate meiotic DSBs, whereas in the *spo11*Δ mutant they are missing. In a positive control, the *com1*Δ mutant, which is defective in DSB repair (29), fragments accumulate as expected. In the *pars11*Δ mutant, DSB-dependent fragments were absent as in the *spo11*Δ mutant (Figure 2C). Therefore, we conclude that Pars11 is required for the formation of Spo11-dependent meiotic DSBs.

### Pars11 protein localizes to early meiotic prophase chromatin prior to DSB formation

**Figure 3.**
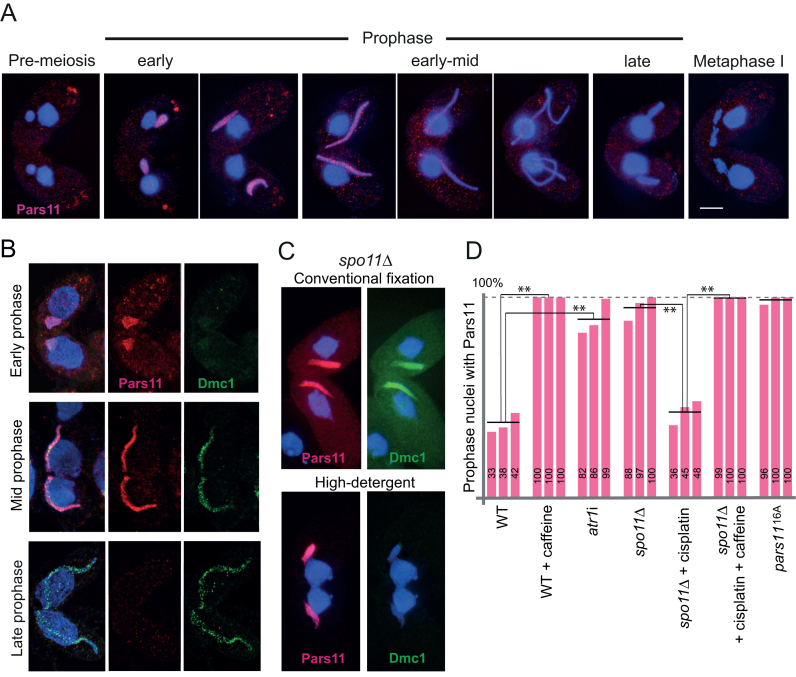
Pars11 localization. (**A**) Pars11 localizes to early meiotic prophase nuclei and disappears prior to full nuclear elongation. (**B**) Localization of Pars11-HA (red) and Dmc1 (green) to chromatin in detergent-spread nuclei in early, mid and late prophase. (**C**) Pars11 localizes to chromatin in *spo11*Δ. Pars11-HA is insensitive to high-detergent fixation, whereas Dmc1 is sensitive. Bar: 10 μm. (**D**) Pars11 localization to prophase nuclei is stabilized in the absence of DSBs (*spo11*Δ), when DSBs are not sensed (ATR depletion by caffeine or *atr1*i) or when Pars11 is not phosphorylated (*pars11*^16A^). Changes in nuclear Pars11 localization are significant (**P < 0.01, paired *t*-tests). Three biological repeats, each with at least 50 nuclei evaluated, were done for each type.

To study the cellular distribution of Pars11, strains expressing Pars11-HA were constructed. In the WT, Pars11 localizes to meiotic nuclei during early prophase and then disappears by the fully elongated mid-prophase stage (Figure 3A). We previously showed that harsh detergent treatment can discriminate between chromatin-associated and soluble nuclear proteins (40). Under these conditions, Pars11-HA is retained in the nuclei; hence, we assume that it is bound to chromatin (Figure 3B). In contrast to Dmc1’s distribution in nuclear foci, which is consistent with its localization to DSBs (40), Pars11 has homogenous nuclear distribution (Figure 3B). Furthermore, Pars11 is expressed before Dmc1 and disappears prior to DSB repair when Dmc1 is still present (Figure 3B).

We next tested whether Pars11 nuclear location is dependent on Spo11 and DSB formation. Pars11-HA was present in the *spo11*Δ mutant after fixation in high-detergent fixative (Figure 3C), indicating that its expression and association with chromatin are independent of DSB formation. (The control, Dmc1, was removed by this fixation method because it is not associated with chromatin in the absence of DSBs; Figure 3C.) Notably, Pars11 persisted for the entire prophase in the absence of Spo11 (and hence DSBs) (Figure 3D). This was reversed by cisplatin-induced DNA damage (Figure 3D). Therefore, it is possible that formation of a sufficient number of DSBs elicits a signal for Pars11 removal from chromatin. We previously showed via chemical inhibition that the sensor kinase ATR (which, in *Tetrahymena*, probably combines the functions of ATM and ATR) is required for the signaling and repair of meiotic DSBs (25). To test whether Pars11 dynamics depend on DSB signaling by ATR, we quantified Pars11 nuclear localization upon ATR inhibition by caffeine and ATR depletion by RNA interference (RNAi). In both cases, Pars11 was stably retained in nuclei (Figure 3D). The same result was obtained following inhibition of ATR-dependent signaling of cisplatin-induced DNA damage in *spo11*Δ (Figure 3D). These experiments indicate that Pars11 nuclear localization is independent of DSBs and suggest that its removal largely depends on the formation and ATR-dependent signaling of DSBs.

### Pars11 is phosphorylated in an ATR-dependent manner

**Figure 4.**
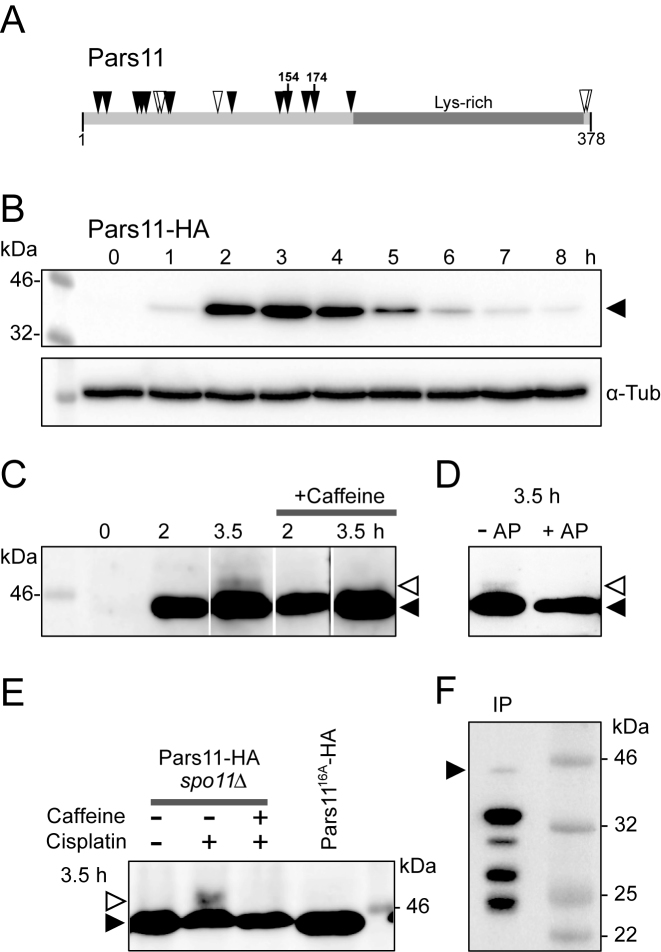
(**A**) Pars11 protein organization. Solid arrowheads: SQ motifs, open arrowheads, TQ motifs. Ser154 and Ser174 are confirmed phosphoepitopes. (**B**) Western blot showing a protein band of the size expected for HA-tagged Pars11 (∼44 kD—solid arrowhead), with maximal intensity at 3 h after induction of meiosis (corresponding to early−mid-prophase) and α-tubulin as the loading control. (**C**) High-resolution SDS-PAGE reveals a caffeine-sensitive (i.e. possibly ATR-dependent) modified Pars11 form (open arrowhead) during late prophase (3.5 h after induction of meiosis). Lanes in panel C were rearranged to improve comprehensibility. The unprocessed gel is shown in Supplementary Figure S4B. (**D**) The modified Pars11 form is sensitive to alkaline phosphatase (AP). (**E**) Pars11 remains non-phosphorylated (solid arrowhead) in *spo11*Δ, but phosphorylation (open arrowhead) is induced by the DNA damaging agents cisplatin, and is suppressed by the ATR inhibitor caffeine. Pars11^16A^ remains also unphosphorylated. (**F**) After immunoprecipitation (IP) most Pars11-HA is degraded (solid arrowhead indicates intact protein).

The 378 amino-acid Pars11 protein (UniProtKB/TrEMBL I7M265_TETTS) consists of numerous N-terminal S/T-Q motifs and a lysine-rich C-terminus (15.8%, compared with an average Lys content of 9.1% for the proteome), which is suggestive of a DNA binding activity (Figure 4A and Supplementary Figure S3). S/T-Q clusters are the preferred phosphotargets of ATM and ATR (42). Two of these sites in Pars11 (Ser154 and Ser174) were in fact confirmed to be phosphorylated in a large-scale phosphoproteomic screen (our unpublished data). Therefore, it is possible that Pars11 becomes phosphorylated during meiotic prophase.

Pars11-HA was detected by western blotting of a meiotic time course of cellular proteins. It became visible at 1 h after the induction of meiosis (corresponding to early prophase) and was most abundant at 3 h (corresponding to early-mid prophase) (Figure 4B). When western blotting was performed after separation on a 7.5% SDS-PAGE gel, an additional weak band appeared at 3.5 h (late prophase, when nuclei lost Pars11 in the corresponding cytological sample) (Figure 4C). The larger band could represent a phosphorylated fraction of the protein. Indeed, this band was sensitive to phosphatase treatment (Figure 4D). Phosphorylation is dependent on Spo11 but was seen in a *spo11*Δ mutant after artificial DNA damage by cisplatin (Figure 4E) or ultraviolet (UV) irradiation (Supplementary Figure S4A). In all cases, the phosphorylated Pars11 fraction was absent after ATR inactivation by caffeine (Figure 4C and E). Together, these observations indicate that ATR is required for the phosphorylation of Pars11 and suggest that Pars11 is removed from mid-prophase nuclei following phosphorylation.

HA-tagged Pars11 was also used for co-immunoprecipitation-coupled and mass spectrometry identification of potential interaction partners. However, the protein turned out to be extremely unstable (Figure 4F) and the only significant hits were several uncharacterized non-meiosis-specific proteins that were not reproducible in two biological repeats (data not shown).

### Meiotic DSBs are overproduced in the absence of ATR

**Figure 5.**
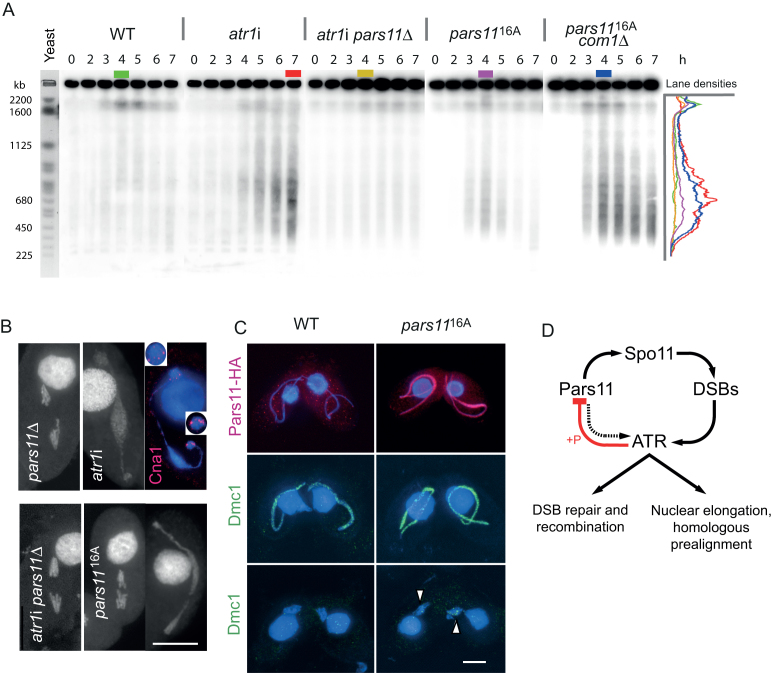
Excessive DSB formation in the absence of ATR and phosphorylatable Pars11 and a model of ATR−Pars11 interaction. (**A**) In the WT, DSB-dependent fragments are transiently formed after onset of meiosis. In the absence of ATR, a few large and numerous smaller fragments indicate excessive DSB formation, which is suppressed by *PARS11* deletion. In the presence of non-phosphorylatable Pars11^16A^, DSB formation is also increased. Increased DSB formation in *pars11*^16A^ is particularly apparent when DSB repair is prevented in a double mutant with *com1*Δ. Density profiles (in arbitrary units) of lanes with the highest intensity (color-coded) show the size distribution of DSB-dependent fragments. Budding yeast chromosomes are shown as size markers. h: hours after induction of meiosis. (**B**) In *pars11*Δ, intact univalents separate in anaphase I. In *atr1*i, a chromatin mass assumes a dumbbell shape during the attempted anaphase. Cna1 staining indicates that kinetochore-containing chromosome parts migrate to opposing ends of the chromatin mass, whereas the bulk of chromatin does not separate (inserts show enlarged kinetochore regions). (Brightness of the somatic nucleus was selectively reduced to improve visualization of the adjacent germline nucleus.) In the *atr1i pars11∆* double mutant, anaphase movement is restored. In the *pars11*^16A^ mutant, effects on anaphase are variable (examples of a normal segregation and a bridge are shown). (**C**) HA-tagged Pars11 has disappeared by late prophase in the WT (see also Figure 4B), whereas HA-tagged Pars11^16A^ persists to late prophase. Dmc1 staining is more intense in *pars11*^16A^ than in the WT, and some Dmc1 foci remain in diakinesis-metaphase I nuclei (arrowheads). Foci in the somatic nucleus arise from antibody cross-reaction with Rad51. Bars: 10 μm. (**D**) Model of DSB control by Pars11 and ATR. Pars11 contributes to DSB-dependent nuclear elongation (dotted arrow). ATR downregulates its own activity via a feedback loop with Pars11 (red).figure at full with

Since Pars11 is required for DSB formation and DSB formation promotes ATR-dependent Pars11 phosphorylation and its removal from chromatin, we investigated whether ATR is involved in regulating DSB formation via Pars11. RNAi-based conditional ATR knockdown strains (in the following designated as *atr1*i) were used to measure meiotic DSB formation in the absence of ATR by the DNA fragmentation assay (Figure 5A). In the WT, most fragments migrated as a single band at around 2000 kb. [Increased resolution due to extended run time suggested a mean fragment size of around 2200–3500 kb—(34)]. However, in the absence of ATR, this band was weak, but a lower molecular-weight DNA smear (∼400–800 kb) appeared from 4 h onward (Figure 5A). This indicates the presence of smaller DNA fragments due to a larger number of DSBs. Density profiles for lanes were produced to compare the size distribution of DNA fragments among the different strains (Figure 5A). DNA fragments were approximately five times smaller in the absence of ATR compared with the WT, indicating that five times as many DSBs are produced. The elevated number of DSBs is not due to the inability to repair DSBs, since smaller fragments are not formed in the *com1*Δ mutant, which also has defective DSB repair (Figure 2C). To make sure that increased DNA fragmentation in *atr1*i is not due to post-meiotic DSBs or to normal autophagic degradation of three of the four meiotic products (34), meiotic stages were checked in all cell samples used for the DNA fragmentation assay. Post-meiotic stages were not found at 6 h and were rare at 7 h (Supplementary Table S2), thus, DNA damage other than meiotic DSBs is unlikely to contribute to DNA fragmentation in *atr1*i.

Additional evidence for the accumulation of unrepaired DSBs was provided by the structure of meiotic chromosomes. In the WT, nuclear elongation at prophase is followed by the formation of five bivalents. In contrast, in the absence of ATR nuclei did not elongate and no distinct condensed chromosome structure could be seen. Only granular masses of chromatin (representing fragmented DNA) were observed (Figure 2C-IV). Occasionally, these assumed a dumbbell shape (Figure 5B). This chromatin arrangement is interpreted as an attempted anaphase I, in which the fragments containing kinetochores are pulled toward the poles but most kinetochore-less fragments remain between the poles. This interpretation was confirmed by the staining for the centromeric protein Cna1, which was found exclusively at the ends of the dumbbell (Figure 5B). In the *atr1*i *pars11*Δdouble mutant, DSBs were not formed (Figure 5A), intact univalents were efficiently restored (in 46 out of 50 metaphase nuclei scored) and nuclei entered anaphase (Figures 1C–V and 5B). Thus, DNA fragmentation in *atr1*i was due to unrepaired meiotic DSBs.

### Excessive meiotic DSBs are produced if Pars11 is not phosphorylated

To directly demonstrate that Pars11 activity is controlled by ATR-dependent phosphorylation, the 16 N-terminal Ser and Thr phosphosites of Pars11 were substituted with Ala to create the non-phosphorylatable version Pars11^16A^ (Figure 4E). In addition, a HA tag was fused to the C-terminus. Meiotic nuclei were found to elongate in *pars11*^16A^, indicating that DSBs were formed and DNA damage signaling was activated (Figure 5C). Notably, Pars11^16A^-HA remained localized to the chromatin of fully elongated nuclei (Figures 3D and 5C) and disappeared only at diakinesis−metaphase I. The persistence of non-phosphorylatable Pars11 confirmed that phosphorylation of WT Pars11 is needed for removal from chromatin at mid-late prophase.

Next, we tested whether more DSBs were formed in the presence of non-phosphorylatable Pars11. The DNA fragmentation assay showed that, as in the *atr1*i mutant, a smear of smaller DNA fragments was formed at the expense of larger fragments (Figure 5A). However, whereas in *atr1*i the intensity of the smear increased with time, in *pars11*^16A^ the smear was weaker and transient. This may be due to asynchronous meiosis in the cell samples, with overlapping DSB generation and repair. Indeed, Dmc1 staining was more intense in late prophase *pars11*^16A^ nuclei compared with WT, suggesting increased DSB repair activity. While the WT had countable Dmc1 foci, the signals merged into large patches in the mutant (Figure 5C), which is another, although non-quantitative, indication of a larger number of DSBs. Only a few Dmc1 foci were retained in diplotene/diakinesis nuclei (at a stage where DNA repair is completed in the WT), indicating delayed or aborted repair (Figure 5C). Practically no viable sexual progeny was produced from *pars11*^16A^ matings [one viable clone from 171 mating pairs—for the viability testing procedure see (28)]. Consistent with the repair of most DSBs, chromosomes or bivalents appeared largely intact in Giemsa-stained metaphase I nuclei (Figure 1C-VI) while anaphases I ranged from seemingly normal to severely affected (Figure 5B). Altogether, this indicates that the DSB repair machinery can largely cope with excessive DSB formation.

To determine the actual extent of DSB formation in the absence of Pars11 phosphorylation, *pars11*^16A^-HA *com1*Δ strains were constructed. DSB repair does not take place in the absence of Com1, which leads to the accumulation of >2000 kb-fragments that are only transiently seen in the WT (Figure 2C). The *pars11*^16A^*com1*Δ double mutant showed an accumulation of smaller fragments, comparable to that seen in the *atr1*i mutant (Figure 5A). This shows that DSB overproduction is similar in the absence of ATR and in the presence of non-phosphorylatable Pars11, suggesting that ATR-dependent DSB restriction occurs exclusively via phosphorylation of Pars11.

## DISCUSSION

### Pars11 regulates the number of DSBs

In *Tetrahymena*, previous examples of genes required for meiotic initiation and DSBs included meiosis-specific cell cycle regulators such as cyclins and CDKs, which act upstream of the process (43,44), but co-factors that support DSB formation by Spo11 have not yet been found. Here, we report that Pars11 has functions in DSB initiation and restriction and in DNA damage sensing.

In the absence of Pars11, DSBs were not formed, suggesting that Pars11 supports the DNA-cleaving activity of Spo11. Since Pars11 localizes to chromatin independently of Spo11, its mode of action might be similar to that proposed for the RMM Spo11-guiding subcomplex of yeast, that is via recruiting Spo11 to the chromosome axis (14). Although a synaptonemal complex and canonical axial elements have not been found in *Tetrahymena*, the sporadic thread-like arrangement of proteins within meiotic nuclei suggests that an underlying axial structure exists (45). Therefore, Spo11 recruitment to the axis may be a requirement for DNA cleavage in *Tetrahymena*, in accordance with the loop-axis model [(14); see (46)]. However, since we could find no homology to any of the RMM factors (see below), the possibility that Pars11 acts in a completely different way, for example by making chromatin accessible for Spo11, cannot be excluded.

The mechanism for sensing meiotic DSBs in *Tetrahymena* is unknown. Although *COM1* (the homolog of yeast *SAE2*) and *MRE11* are part of the DSB processing machinery, they cannot be part of the DSB signaling pathway because DSBs are signaled in their absence (29). *Tetrahymena* possesses only ATR (25,47), which means either that this sensor kinase is capable of signaling unprocessed DSBs (as normally done by ATM) or that the initial signal triggering strand resection must be created by another sensor kinase. The mediator and effector phosphorylation cascade downstream of ATR is unknown but ends with the polymerization of intranuclear microtubules that elongate the prophase nuclei and with activation of the homologous recombination machinery. Here, we found that nuclear elongation following artificial DNA damage was reduced in the absence of Pars11, suggesting that the signaling of artificial DNA lesions was attenuated. Therefore, Pars11 promotes either the sensing of DNA damage or the DNA damage response. It is unclear whether either of these actions happens via the processing of DNA lesions to make them better substrates for recognition or via activating another DNA damage response pathway.

At last, Pars11 negatively controls DSB formation. In the absence of ATR, the number of DSBs is increased by an estimated five to six times compared with WT. The smear of DNA fragments had the same size range in both the *pars11*^16A^ and *atr1*i mutants (Figure 5). However, the smear of fragments is less intense and transient in the *pars11*^16A^ mutant compared with the *atr1*i mutant, in which the repair machinery is not activated and cells arrest with unrepaired DSBs. The transient nature of DSB-dependent fragments, the largely intact bivalents/chromosomes at diakinesis-metaphase I and the only moderate effect on anaphase I in the *pars11*^16A^ mutant indicate that, surprisingly, the repair machinery can cope with the additional DSBs. To prevent DSB repair, a *pars11*^16A^*com1*Δ double mutant was created. This showed that the extent of DNA fragmentation and the number of DSBs are similar in the *atr1*i and *pars11*^16A^ mutants (Figure 5). Since, based on the cytological analysis of Dmc1 foci, the number of DSBs is estimated to be close to 200 in the WT (48), the number in the *atr1*i and *pars11*^16A^ mutants may be ∼1000. While this estimate may be influenced by many imponderables, such as the unknown proportion of fragments in the WT that are too big to enter the gel, a 5 to 6-fold rise in the DSB number is comparable to the rise in a mouse *atm*^−/−^ mutant, in which a >10-fold rise in covalent Spo11-oligonucleotide complexes suggested a corresponding increase in DSBs (49).

### A conserved mechanism for DSB control

Meiotic DSBs produce free DNA ends that are required for homology recognition and recombination. Although the factors that induce DSBs are present in excess (13), the number of DSBs is limited to <300 in most organisms [see (11,50)]. Several strategies by which organisms negatively control meiotic DSB formation at the local and global levels have been described (51): a global, genome-wide DSB control is achieved by synaptonemal complex formation, which signals successful homologous recognition and pairing to the DSB-forming machinery to stop it working. Synapsis-dependent DSB control was found in mice, *Caenorhabditis elegans*, and budding yeast (50,52–54). In addition, a limited temporal window at meiotic prophase during which Spo11 and its partners are expressed may contribute to global DSB control (50). At the local level, some sort of interference, whereby early DSBs influence whether and where later DSBs are formed, may prevent repeated DNA cleavage in the same region (50,55). Studies in the mouse, *Drosophila*, budding yeast and *Arabidopsis* have identified ATM/Tel1-dependent control of DSB formation [(49,55–60), Kurzbauer and Schlögelhofer, pers. commun.; for a review see (21)]. Furthermore, altered DSB distribution in yeast and mice may be caused by the occupation of less amenable chromatin substrates by surplus DSBs (49,58,60).

In budding yeast, a complex of nine proteins is required for DSB formation by Spo11 (61). Three of them, Rec114, Mer2 and Mei4 form the RMM subcomplex, which assembles on chromatin once replication has taken place and which is required for Spo11 binding to sites of DNA cleavage (14). Among them, Rec114 has numerous ATM/ATR-dependent phosphorylation sites. Carballo *et al.* (50) provided evidence for phosphorylation of Rec114 by ATM/Tel1 and ATR/Mec1 as one mechanism for DSB downregulation in addition to Rec114 degradation upon activation of Ndt80 (a meiosis-specific transcription factor) and its removal from chromatin during homologous synapsis. Phosphomimetic mutations at Rec114 S/T-Q sites prolonged the Rec114 axis association, delayed synaptonemal complex formation, and strongly reduced the DSB number; in contrast, non-phosphorylatable amino acid substitutions moderately increased DSBs, as measured by the abundance of Spo11–oligo complexes (as a readout for DSB formation) and by other DSB markers (50). The authors concluded that Rec114 phosphorylation reduces both its interaction with DSB hotspots and DSB formation. However, Mohibullah and Keeney (58) found that the increase in Spo11–oligo complexes was much lower in a non-phosphorylatable *rec114* mutant than in *tel1/atm* null or kinase-inactive mutants. Therefore, they argued that other phosphotargets such as Hop1 must contribute to Tel1/ATM-mediated DSB suppression in budding yeast [see (21)].

DSB-2, a protein that promotes DSB formation in *C. elegans*, was found to have characteristics similar to Rec114: It localizes to chromatin during the period of DSB formation independently of SPO-11; it disappears once DSBs are processed, and its residence is prolonged in mutants, which are unable to create CO recombination intermediates (4). DSB-2 (together with its paralog DSB-1) was proposed to act by creating a DSB-permissive chromatin structure or by recruiting and/or activating SPO-11. The persistence of DSB-2 for as long as DSBs need to be formed and its disappearance when sufficient CO intermediates have been formed led the authors to speculate that DSB-2 is part of a negative feedback system like the one proposed for Rec114. This supposition gained support from the later finding that DSB-2 is a distant homolog of Rec114 homolog (62). Distant homologs of Rec114 in all higher eukaryotic groups were recognized by the presence of conserved short signature sequence motifs (62,63).

MEI4 and REC114, members of a putative mouse RMM-like subcomplex, have similar behavior to yeast Rec114 in that their association to chromosome axes is required for DSB formation, and they disappear in parallel with DSB repair and synapsis (64,65). Therefore, it was speculated that loss of this association upon DSB repair could contribute to switching off meiotic DSB formation (64).

Together, these observations suggest that a substantial contribution of Rec114 orthologs to DSB regulation, most likely governed by phosphorylation status, is shared by plants, animals and fungi.

### DSB control in *Tetrahymena* may be similar but not identical

The protist *Tetrahymena* is evolutionarily distant from the common meiotic model organisms; thus, the existence of a common DSB regulatory mechanism in this species may indicate the deep evolutionary conservation of this process. The role of Pars11 in DSB control resembles that of Rec114. Pars11 localizes to chromatin and allows Spo11 to induce DSBs. Once a threshold number is reached, the DSBs activate ATR, which causes elongation of the nucleus (thereby promoting homologous pairing) and activates DSB repair. At the same time, ATR (directly or indirectly) phosphorylates Pars11, leading to its inactivation by removal from chromatin or degradation in late prophase, thereby terminating DSB formation. Thus, Pars11 inactivation, in turn, terminates ATR activity (via indirect ATR self-regulation) and thereby allows meiosis to progress from prophase (Figure 5D).

Despite the similar roles of Pars11 and Rec114 in DSB control, a conserved motif search in Pars11 using MEME (66) did not reveal the diagnostic signature sequence motifs or other motifs of any Rec114 family or the other RMM complex members (Supplementary Figures S5 and 6). Another possibility would be a relatedness of Pars11 to the axial element protein Hop1. Hop1 shows features that suggest its involvement in synapsis-dependent DSB control in budding yeast: it is required for efficient DSB formation (67), it is phosphorylated by ATM/ATR in a DSB-dependent manner and it is removed once DSBs are formed (68). However, no similarity exists between Pars11 and yeast Hop1 (Supplementary Figure S6). Moreover, while Pars11 is fairly well conserved among *Tetrahymena* species (Supplementary Figure S7), it does not share recognizable similarity to any protein in other lineages, not even within the ciliates, which indicates its rapid divergence. Therefore, it is possible that Pars11 may have diverged beyond recognition from a common ancestral Rec114-, Mei4- or Hop1-related protein. Alternatively, Pars11 may be an orphan protein that has been harnessed for a similar purpose.

Unlike in yeast, where Rec114 phosphorylation alone may not account for Tel1/ATM-mediated DSB suppression, the occurrence of similar numbers of DSBs in *atr1*i and *pars11*^16A^ suggest that Pars11 is the only relevant ATR-phosphotarget. Of course, DSB formation may also be restricted by the limited time window during which Spo11-dependent DSBs can be created since even non-phosphorylatable Pars11 eventually disappears from the nucleus at the end of prophase. Nevertheless, in the absence of an SC and hence sophisticated synapsis-dependent DSB control, *Tetrahymena* meiosis, which in general employs a sparse repertoire of genes (27), may rely on an ATR−Pars11-dependent mechanism as the major, if not the only, DSB regulatory mechanism.

## Supplementary Material

Supplementary Data
