# Supplementary material for: A chromatin-associated protein required for inducing and limiting meiotic DNA double-strand break formation
Source: Nucleic Acids Res. 2018 Oct 24;46(22):11822–34. doi: 10.1093/nar/gky968 (PMC6294514; doi:10.1093/nar/gky968)
Supplement: Supplementary Data [file gky968_supplemental_files.pdf]

Table S1. List of primers

Purpose	Primer	Sequence (5 → 3', Adaptors used for Gibson assembly are labeled in red)
PARS11 knockout		
	Pars11_5UTRf1199_Not1	AGTTCTAGAGCGGCCGCCTTCTTTGCATTTATAAGATACAAG
	Pars11_5UTRr1862_N4	GTCAGGTGCCTGGTACCCTTTCTTACTTCTGATTTTTTTAATC
	Pars11_3UTRf3159_N4	CTGACGTCGCACCATCCCTTTTTAGTTATATTTTTGTGGATTG
	Pars11_3UTRr3801_Not1	ACCGCGGTGGCGGCCGCTTACAAAGCTATTAAATTGTGATTC
Pars11 C-terminal HA tagging		
	Pars11_CDSf2468_Not1	AGTTCTAGAGCGGCCGCATCAAGGCTAAATCATCCTAATC
	Pars11_CDSr3125_HA	GTCGGGGACGTCGTAGGGGTATCCATTATTTTAAGTTTAGGTTGCCTTT
	Pars11_3UTRf3126_HA	CCCTACGACGTCCCCGACTACGCCTGAATTTTAATATCAATTATTTTTT
	Pars11_3UTRr3569_N4	GTCAGGTGCCTGGTACCCAATTCATATTAATAAAGGAAATTTG
	Pars11_3UTRf3564_N4	CTGACGTCGCACCATCCCTGAATTATAAATTCTAGATTTCTAAT
	Pars11_3UTRr4151_Not1	ACCGCGGTGGCGGCCGCAATAGCATTCACAATATACTTTTG
PARS11 knockout validation		
	Pars11_Cdsqf2409	CAAGAAAGTAAGGTCTCTTAATTTG
	Pars11_Cdsqr2745	TAATTACATTGCTCTAGAAAAAGTG
ATR1 RNAi		
	ATR1_N5if7625_5F	TAAACTTAAACATCCCGGGGGATCCATGAGCTAGATTAAACACTTTTTAG
	ATR1_N5ir8282_5R	TTGCATATCCGTTACTTACGGATCCTTAATTAGTTTCATTATGATCCTTG
	ATR1_N5if7625_3F	GCTGACCGATTCAGTTCGCCTGCAGATGAGCTAGATTAAACACTTTTTAG
	ATR1_N5ir8282_3R	TAAAAGAAGAATTCAAAGGCTGCAGTTAATTAGTTTCATTATGATCCTTG
Pars11-16A construct		
	Pars11_16AQ_5UTRr912	CTTCTTATTGATATATGTTTTCAGACTCAT
	Pars11_16AQ_IDTf	ATGAGTCTGAAAACATATATCAATAAGAAG
	Pars11_16AQ_IDTr	TAAGCAATTTGTCCAACCATCAA
	Pars11_16AQCDSf1514	CTTGATGGTTGGACAAATTGCT
RT-PCR		
	18s F	CCTGGGAAGGTACGGGTAAT
	18s R	AAGGTTCACCAGACCATTCG

RT-PCR		
	Spo11_cds2f2319	GCTAAAGGTTTAGTAACTGGTAATG
	Spo11_cds2r2668	TTTCCCAGTAATCATTATACAATTC
Sequence for *PARS11* 1-603 bp mutation		
		atg agt ctg aaa aca tat atc aat aag aag aaa gct taa aac tta ggt tct gct tag tca tcg gct gca gct ctc aca tca ctt tag tca aat tgc ttt aca gca aag gga taa atg gtt gct taa aat gct tag aac gct cag aat acc att caa atg cag tgc aat gct caa gct cag tag ata ctc gct caa ggt att tat ttt aat agt tca att tgt aat taa ttt aaa tat tta atc tct cag ctg ctt aat ttc ctg tgc cta aaa act cta gcc tta tga ttg aag aac caa ata tct ttg gat aaa ctg gat aca gct cat tca aaa ata aga acc taa att ctg ctt aga ata cat cct ttt taa att agt tag ctt aag acg aag cta atg att ctt aca att aaa ttc tac atg aga ata att tta ttt aaa atc cta atc tga act aaa gct ttc taa att aaa gct atc aac ttc ttg ctt agc ata acc taa agt aag ctc aat tgt ttc ttc cag cga tcc aag aaa gta agg tcg ctt aat ttg atg gta agt ctg ctt aga tat tta atc ctt ctg aat caa ggc taa atc atc cta atc tga tat aaa gta gct tga tgg ttg gac aaa ttg ct

Table S2

Frequency distribution of cytological stages in samples taken from *atr1*i cultures for PFGE from t=5 h, 6 h and 7 h after meiosis induction. Post-meiotic stages were largely absent at these timepoints. Therefore, post-meiotic DSBs cannot acount for the DNA fragmentation seen.

t	Stages %	Pre-meiotic	Early prophase	Mid prophase	Late prophase –metaphase I	Attempted Anaphase I	Post-anaphase	RNAi escapers (WT-like stages)	Sample size (no. of nuclei)
5 h		15.5	50	35.5	5	0	0	3	200
6 h		10.5	10	11.5	58	8	0.5	1.5	200
7 h		11	3	6	50	7	21	2	250

Prophase stages were classified as early mid and late according to the increasing degree of chromosome condensation, since the length of nuclei does not change in the absence of ATR.

Table S3

BLAST search for Pars11 protein homologs

Group		Species	Protein ID	BLASTP E-value*
Multicellular animals		*Homo sapiens*	sp|Q9Y4D8-4|HECD4_HUMAN	0.21
		Mus musculus	sp|Q8CAQ8-5|MIC60_MOUSE	0.12
		Danio rerio	ENSDARP00000122296	0.021
		Drosophila melanogaster	FBpp0082802	0.42
		Caenorhabditis elegans	ZK430.1	3.3
Land plant		*Aradidopsis thaliana*	AT5G45610.1	0.95
Fungi		*Saccharomyces cerevisiae*	YOR237W	0.12
		Schizosaccharomyces pombe	SPAC664.14.1:pep	0.52
Protist	Tetrahymena	*Tetrahymena borealis*	EI9_05736.1	2.31E-56
		Tetrahymena elliotti	EI7_06775.1	1.79E-58
		Tetrahymena malaccensis	EIA_05075.1	0
	Other ciliates	*Paramecium tetraurelia*	GSPATP00036354001	0.065
		Ichthyophthirius multifilis	IMG5_094660	0.17
		Oxytricha trifallax	Contig20793.0.g60|Guanylate-binding	0.21
		Stylonychia lemnae	Contig10830.g11578	0.001
	Other protists	*Plasmodium falciparum*	PF3D7_1227500	0.45
		Toxoplasma gondii	TGME49_206430	0.61
		Trypanosoma brucei	Tbg972.7.3660	0.57
		Giardia lamblia	GL50581_1183	1.9

*BLASTP e-value threshold was set to 10 (default).

Figure S1

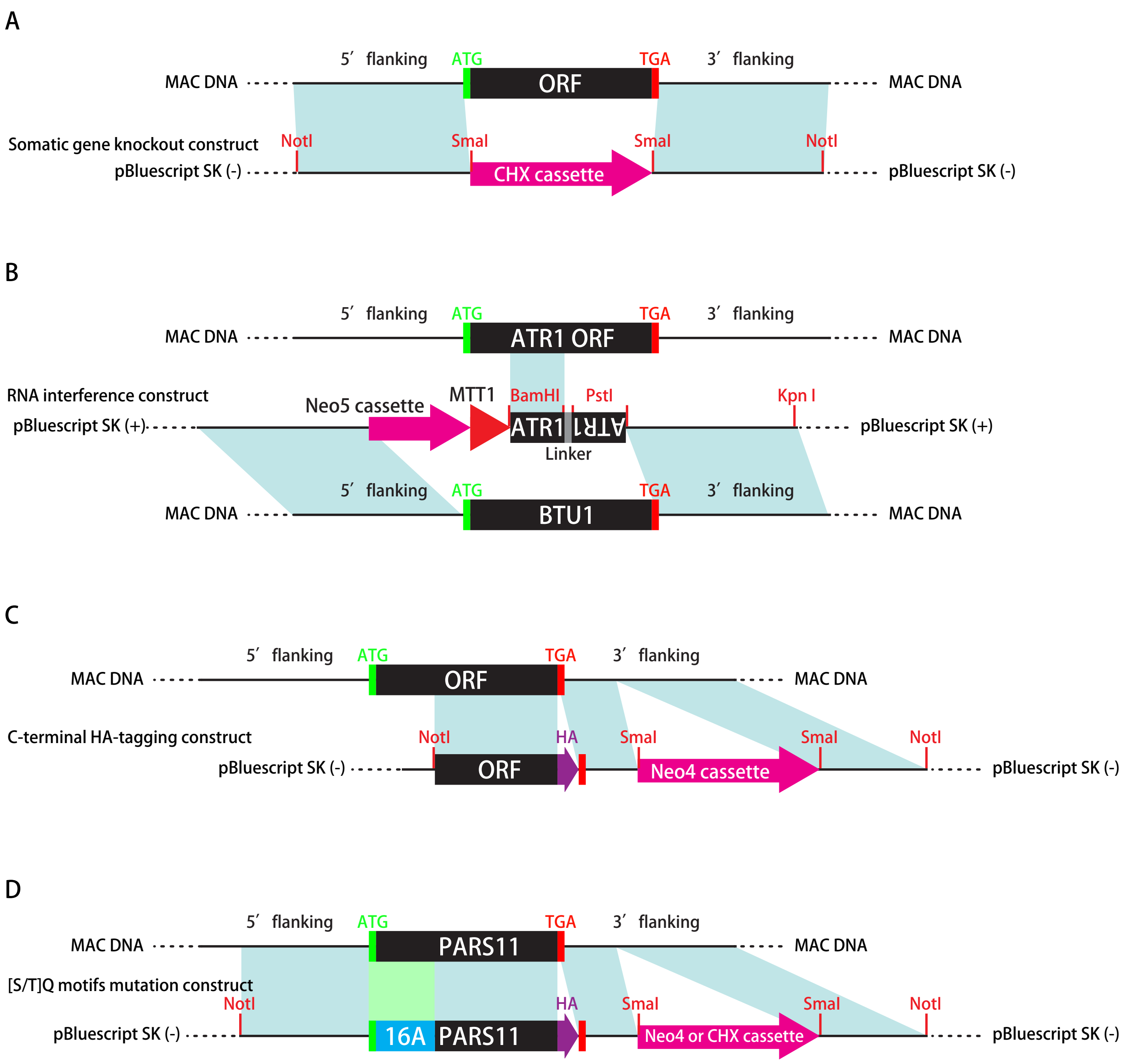

A. Diagram of the somatic gene knockout construct. DNA fragments used for homologous recombination were amplified from wild-type Tetrahymena genomic DNA using PCR. The NEO4 (paramomycin resistance) or CHX (cycloheximide resistance) cassette was removed from the respective plasmids by SmaI digestion (Gao et al. 2013, Genes Dev. 27: 1662-1679). DNA fragments and drug resistance cassette were then cloned into the linearized pBluescript SK(-) vector by Gibson assembly. DNA fragments in the knockout construct and their corresponding loci in the somatic genome are indicated by light blue fields.

B. Diagram of the ATR1 RNA-interference construct. A fragment of the ATR1 ORF (from 1295 bp to 1952 bp) was amplified from genomic DNA using PCR. Two copies of this fragment were inserted end-to-end behind a Cd2+-inducible MTT1 metallothionein promoter (red arrowhead) in plasmid pNeo5-RNAi (Akematsu et al. 2017, eLife 6: e26176). After shooting the linearized plasmid DNA into Tetrahymena cells, the NEO5 (paramomycin resistance) cassette and ATR1 RNA-interference cassette is integrated into the nonessential BTU1 gene locus due to flanking homologies.

C. Diagram of the C-terminal epitope tagging construct. The Pars11 C-terminal HA tagging construct was generated in a similar way to the PARS11 somatic knockout construct.

D. Diagram of the construct for the substitution of Pars11 N-terminal S/T-Q sites. This construct was generated in a similar way as the PARS11 somatic knockout construct. In this construct, all 16 serine and threonine codons within the 603 bp 5´ PARS11 coding sequence were replaced by alanine codons (16A, blue box).

Figure S2

PARS11 knockout confirmation using RT-PCR

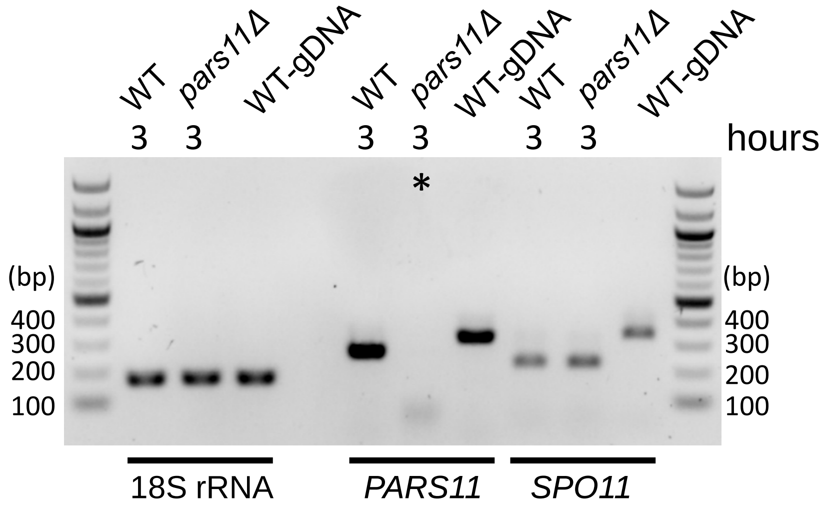

Size				
	cDNA	178 bp	271 bp	234 bp
	gDNA	178 bp	337 bp	350 bp

To confirm the deletion of the somatic *PARS11* gene in *pars11Δ* strains, mutants of different mating types were starved and mixed to induce conjugation. Total RNA of *pars11Δ* mating pairs was isolated at 3 hours after induction of meiosis using the TriFast reagent (PeqLab, Erlangen, Germany). cDNA was then synthesized using 1 µg of DNase I treated total RNA with the RevertAid H Minus Reverse Transcriptase kit (Thermo Fisher Scientific, Waltham, MA, USA) and random hexamers (Integrated DNA Technologies, Leuven, Belgium). cDNA synthesized from total RNA isolated from wild type 3 h after induction of meiosis, and genomic DNA (gDNA) extracted from vegetative cells of wild-type strain B2086 were used as controls. *PARS11* fragments were amplified from WT cDNA and gDNA samples using the PARS11-specific intron-spanning primers whereas this fragment was not amplified using *pars11Δ* cDNA (see asterisk). This result, together with the gDNA qPCR result (not shown), indicates the knockout of the *PARS11* gene from the MAC genome of *pars11Δ* mutants. *18S* rRNA/rDNA and *SPO11* specific fragments were amplified as loading control and indicator of meiosis, respectively. Primers used for PCR reactions are listed in Table S3.

Figure S3

Predicted secondary structure of the Pars11 protein

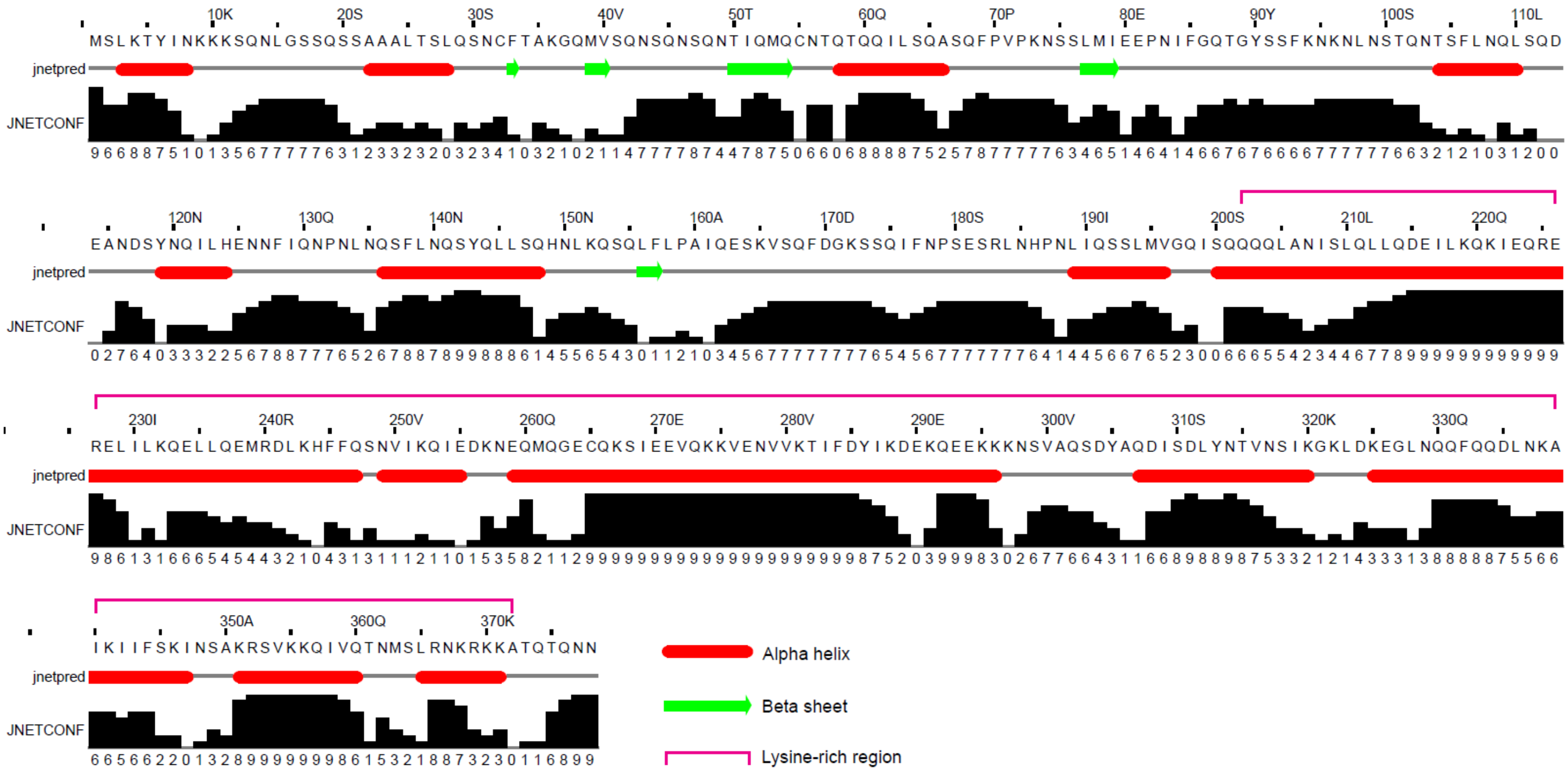

The N-terminus of Pars11 is poor in secondary structural elements, but many alpha helices are found in its C-terminal lysine-rich region. The secondary structure of the *T. thermophila* Pars11 protein was predicted by JPred4 with default settings (Drozdetskiy et al. 2015, Nucl. Acids Res. 43: W389-W394). Putative alpha helices and beta sheets are annotated as red bars and green arrows, respectively. The confidence estimate for the prediction is indicated by both the height of the black bars below the structures and the number. High values mean high confidence levels.

Figure S4

a

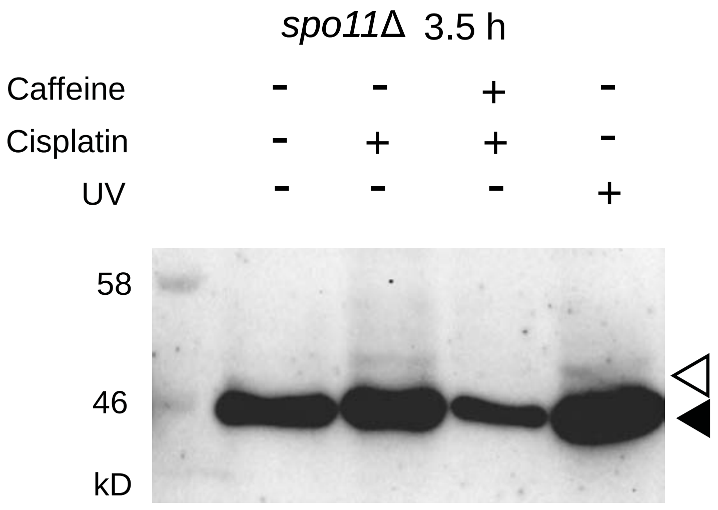

Pars11 remains nonphosphorylated (solid arrowhead) in *spo11*Δ, but phosphorylation (open arrowhead) is induced by DNA damaging agents cisplatin and UV, and is suppressed by the ATR inhibitor caffeine

b Original version of the gel shown in Figure 4c

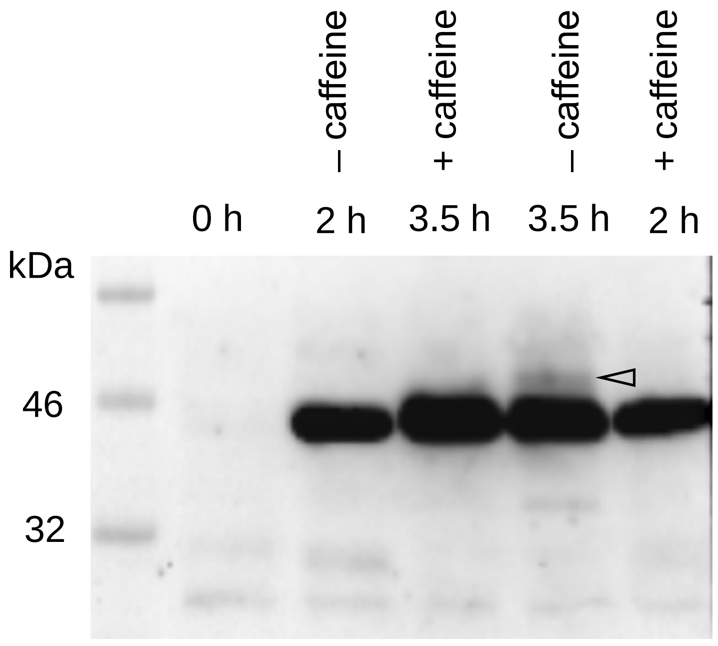

Western blot of HA-tagged Pars11 (~44 kDa). A caffeine-sensitive (i.e. possibly ATR-dependent) modified Pars11 form (open arrowhead) is present during late prophase (3.5 h after induction of meiosis).

Figure S5

MEME search for shared motifs in Pars11 and the Rec114 family

```
>Tetra_Pars11
MSLKTYINKKKSQNLGSSQSSAAALTSLQSNCFTAKGQMVSQNSQNSQNTIQMQCNTQTQQI
LSQASQFPVPKNSSLMIEEPNIFGQTGYSSFKNKNLNSTQNTSFLNQLSQDEANDSYNQILH
ENNFIQNPNLNQSFLNQSYQLLSQHNLKQSQLFLPAIQESKVSQFDGKSSQIFNPSESRLNH
PNLIQSSLMVGQISQQQQLANISLQLLQDEILKQKIEQRERELILKQELLQEMRDLKHFFQS
NVIKQIEDKNEQMQGECQKSIEEVQKKVENVVKTIFDYIKDEKQEEKKKNSVAQSDYAQDIS
DLYNTVNSIKGKLDKEGLNQQFQQDLNKAIKIIFSKINSAKRSVKKQIVQTNMSLRNKRKKA
TQTQNN

>Yeast_Rec114
MYEYCSVVIKKYSKYTIPSFAPNGFQSMLEPPQIDKWQHLSANCTLQFRVLLMDSRQILINV
VLNNSTLLENIRLPLGDNQDLIQFSCKSPIISCKYISEEFGPRMLRRFQMNLPNDVEFNRTV
VSLKNLNFVLRTARTSIAQSTITSQVQGNNNGTKVCFTEGPKVSSYTNPNTQFQTQNMIMDF
SQRYQEESERESNNRSNITLPHDSIQIAQQIWPNTDLNVVQSSQDLNTPMATQTVLGRPESL
IVQPLEVSQSPPNTTNCLPNAENKKKKVDTTSDFTSRKEIALCKTGLLETIHIPKERESQMQ
SVTGLDATPTIIWSPGKDNTAKKNTSNKKNIDDKLTNPQKSGNTHTPDRNKEVLPNGTLNET
RKEASPSEGLTIRVKNVNRNASRKISKRLIKEKLKDEEFMKWVNKVETVLNKMFEK

>Elegans_DSB-2
MSARGLKVEMYYKLAEKNEAETSDVSILIDGHKRKFAVICNGIGREMVDLHYDSKKFPRIFS
RERHLYVMFSESTSDGFRLTFGATEREQFLRIMHSLGCLLDTAIVKFSQQSSSSQPPPMRRD
STAVFNNSYNQYTSFQQASQPRVFSTIKLELSGDSCNSAFQPSQPTGFPDNQLLTIKKSSSN
QISLSVEVADKCIQTEDLVDLLADDEYCMRKALRKMIENPSFIKAVAAAENTLQYIPQAEQE
GLEDVTEPPINDIFFDRFHQPQLEQHDSVPED

>pombe_rec7
MNIFPIVKYSVAEDSYTTDSSHINWSHYSDGGFTLSLTSGLLQIRQHEELIQSINLLDLWHQ
LIPGTKEKCLTLLSRAPCMNIRAFTNNVMKRFQVKFPSDVHYMKAKVEFEKLGLVFKDAKSS
SEKKQFNNSQSQSNNSQELSLMNNAYNKSSAQQPNLLLQPSYIPMTQTATTAVNNSTNYVNP
APLQHVMPNAEIFSNTPPLKRFRGDAGMTQMPLRSDTSIESITASQQPTWDENVVITSSPFN
PNRNAYSYGANSQYPIIAATPLNSQTQASWVAQPENQAYANLIPSPPTTSQILPTELTEEEK
QLRSKVLFYLKQDSFIQLCQSLERVWNKM*

>Mus_rec114
MSEAGNVASGLGLPGEVSQWSLKRYGRFMLLDNVGSPGPSSEAAAAGSPTWKVFESSEESGS
LVLTIVVSGHFFISQGQTLLEGFSLIGSKNWLKIVRRMDCLLFGTTIKNKSRMFRVQFSGES
KEEALERCCGCVQTLAQYVTVQEPDSTTQELQQSQGPREAGESQGKDPLQQGPSLTLEQHVC
MAAGAGVLQERTSVTHRAQSILAPEKLTLAYEGSSWGTEELGPFLRLCLMDQNFPAFVEEVE
KELKKITGLRN

>Maize_Phs1
MRGVHRHQGTWIPASCPASLCVCHPSLPSAVPVLTISIGDVVFEEHFVSILNFSWPQVTCVT
QCPIRGSRVVFVSFCDKFKQIQKFAVRFPQPCDAESFLSCVECSCGSSGTMDIIPFGSDYVC
EDSSASEYIVSNGLHHRLDDASNLEEQCFDHTIDEPPMNYHEETDQHVLEPLSASNTSNNSA
FPPSFNQMLKSCSIDYDQEEPCPLAASNHVLQEVYVLDTSHDERTAGKGMDAAEGVDASILT
YDLMARIKTYMADESFNDMLLKLDKAIDELGGDMSL
```

Discovered Motifs

1.

***E*-value:** 7.6e+001 **Site Count:** 2 **Width:** 6

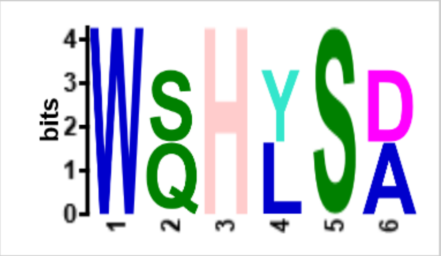

Log Likelihood Ratio: 35 **Information Content:** 22.9 **Relative Entropy:** 25.3 **Bayes Threshold:** 9.91961

Name	Start	*p*-value	Sites
4. pombe_rec7	25	2.86e-9	SYTTDSSHIN WSHYSD GGFTLSLTSG
2. Yeast_Rec114	37	3.64e-8	SMLEPPQIDK WQHLSA NCTLQFRVLL

2.

***E*-value:** 8.5e+001 **Site Count:** 2 **Width:** 9

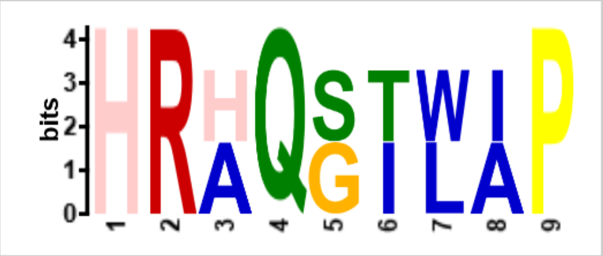

Log Likelihood Ratio: 50 **Information Content:** 33.9 **Relative Entropy:** 36.2 **Bayes Threshold:** 9.90614

Name	Start	*p*-value	Sites
6. Maize_Phs1	5	1.34e-13	MRGV HRHQGTWIP ASCPASLCVC
5. Mus_rec114	202	1.11e-9	GVLQERTSVT HRAQSILAP EKLTLAYEGS

3.

***E*-value:** 8.8e+001 **Site Count:** 2 **Width:** 6

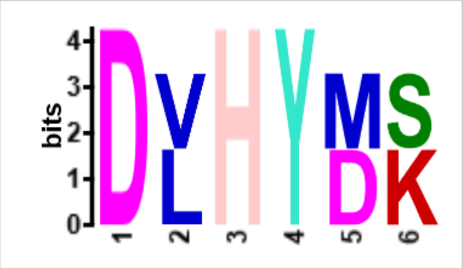

Log Likelihood Ratio: 35 **Information Content:** 22.9 **Relative Entropy:** 25.1 **Bayes Threshold:** 9.91961

Name	Start	*p*-value	Sites
4. pombe_rec7	101	4.26e-9	MKRFQVKFPS DVHYMK AKVEFEKLGL
3. Elegans_DSB-2	49	3.17e-8	ICNGIGREMV DLHYDS KKFPRIFSRE

4.

***E*-value:** 1.7e+002 **Site Count:** 2 **Width:** 8

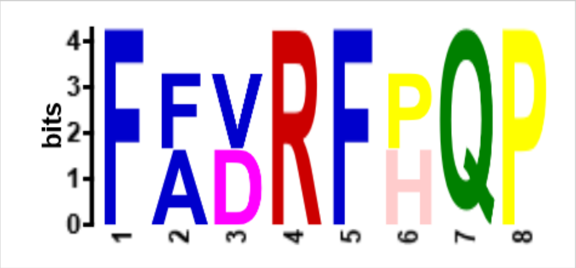

Log Likelihood Ratio: 45 **Information Content:** 31.6 **Relative Entropy:** 32.7 **Bayes Threshold:** 9.91064

Name	Start	*p*-value	Sites
3. Elegans_DSB-2	262	9.08e-12	DVTEPPINDI FFDRFHQP QLEQHDSVPE
6. Maize_Phs1	86	1.86e-10	FCDKFKQIQK FAVRFPQP CDAESFLSCV

5.

***E*-value:** 1.7e+002 **Site Count:** 2 **Width:** 8

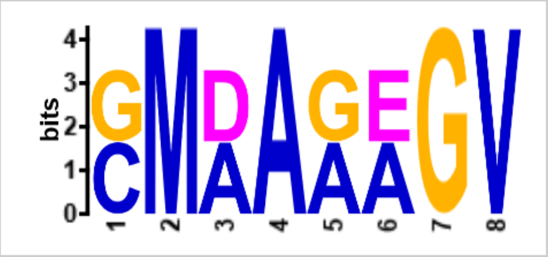

Log Likelihood Ratio: 45 **Information Content:** 30.6 **Relative Entropy:** 32.6 **Bayes Threshold:** 9.91064

Name	Start	*p*-value	Sites		
5. Mus_rec114	186	6.14e-11	GPSLTLEQHV	CMAAGAGV	LQERTSVTHR
6. Maize_Phs1	235	8.22e-11	TSHDERTAGK	GMDAAEGV	DASILTYDLM

6.

***E*-value:** 2.0e+002 **Site Count:** 2 **Width:** 14

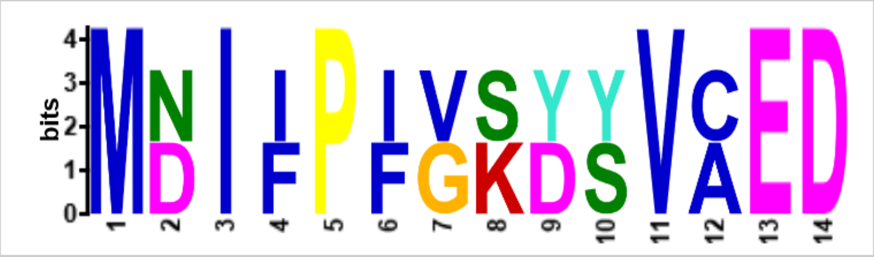

Log Likelihood Ratio: 74 **Information Content:** 52.5 **Relative Entropy:** 53.6 **Bayes Threshold:** 9.88341

Name	Start	*p*-value	Sites		
6. Maize_Phs1	113	1.39e-18	VECSCGSSGT	MDIIPFGSDYVCED	SSASEYIVSN
4. pombe_rec7	1	4.31e-15		MNIFPIVKYSVAED	SYTTDSSHIN

MOTIF LOCATIONS

○ Only Motif Sites ○ Motif Sites+Scanned Sites ◉ All Sequences

Name	*p*-value	Motif Location
1. Tetra_Spop1	8.76e-1	
2. Yeast_Rec114	2.68e-3	
3. Elegans_DSB-2	2.68e-10	
4. pombe_rec7	1.93e-19	
5. Mus_rec114	6.87e-10	
6. Maize_Phs1	2.72e-34	

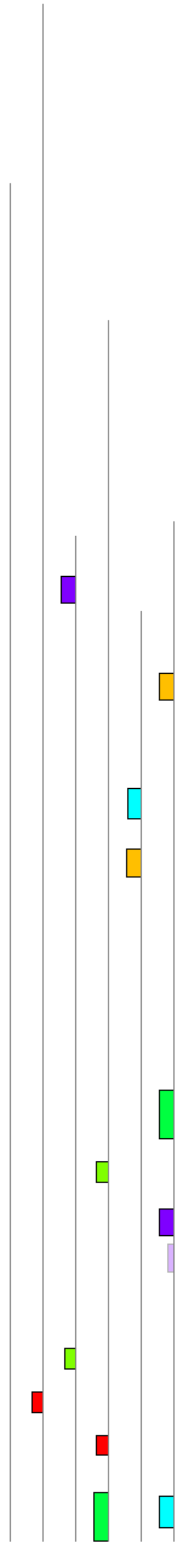

MEME search for shared motifs shared in Pars11 and the Mei4 family

```
>Tetra_Pars11
MSLKTYINKKKSQNLGSSQSSAAALTSLQSNCFTAKGQMVSQNSQNSQNTIQMQCNTQTQQILSQ
ASQFPVPKNSSLMIEEPNIFGQTGYSSFKNKNLNSTQNTSFLNQLSQDEANDSYNQILHENNFIQ
NPNLNQSFLNQSYQLLSQHNLKQSQLFLPAIQESKVSQFDGKSSQIFNPSESRLNHPNLIQSSLM
VGQISQQQQLANISLQLLQDEILKQKIEQRERELILKQELLQEMRDLKHFFQSNVIKQIEDKNEQ
MQGECQKSIEEVQKKVENVVKTIFDYIKDEKQEEKKKNSVAQSDYAQDISDLYNTVNSIKGKLDK
EGLNQQFQQDLNKAIKIIFSKINSAKRSVKKQIVQTNMSLRNKRKKATQTQNN

>Yeast_Mei4
MSRGKLEDMEQKETSEVDWIICFALIQSRNPTLWKRALSRKKGDVEDVGALKSEKNLKINPRENS
KHIYKWVAPFENGFLNNKSLFAHLEPIYNFLCQNKYKSFEDAVGLKELQSFSKDVSTADINNWFL
PRYKILLKILSLKTKEIDFRGLSQVFQTLQILLVSHYSHRIDSDSSFKRTLIDVHVFNFIAKFLF
NRILLKKNQNDPKWLQNFYDQGDGKHLCDKVDYKRLCSLHFTLIYSIINIQLIKIKTNQTFEPQI
LKYVSVLKLIEHILIIIESLIHVLIRFVSKHKLICINRKKAYCRVYLERELSLKKTYLKNFYSVI
SGVPEKELGGLLKILKIVILSLLETFESIEWQHLKPFLEKFPAHEISLQKKRKYIQAALLITAER
NLIARFRLSRWFNETENI

>pombe_rec24
MNGTNTEDNSKQILIQTMYTYDSSGETLKIAIAWKIILKKPKGKNIKDYIEALRKGIEDQEHCEK
YASTLLEPRPKTKKDVVLKNSNVTECVALKAKPFSKKIEDMDIFLLTNVHENLQEKRQTSGSLAH
LDIEYTFNGLFRFLKCTADIKLKQTKVYEGADFLRIKTLFEEIFMFLKRDCKSPLVLTRLVELGD
YVLDLIIITQSIMQNNANNGTGVISRAKFLEFYVFLEQLIFNKLSFASVEQLEKLLDQIVKRMKI
CFTYCKNDNPSIRLLYSECFFSYAEIYFPCLHSFDAQLSSAASKCVQILRDIITNEELQTDKQEL
SKSAYSAPSILLIGLKDMLFPEDIS

>Mus_MEI4
MDIQPWYLKTSKLALALAIIHSKPADRSSREYTEYLASLVTQKESTWKSKLEALEAEVLQLRQKL
LLSRISSGLFKNGPDVLPTLSDQEPTSSENTLTLMDDSGCVLSNEQRNEPAELSQHFVESTDPPL
LPLPLEKRPRTTLENPLSSHMQFFQHLLELKKWTESSSLKVYLTHFEKDSSTVSDSVSQLLDALI
TFYRNPKLPFSSFWTEAVGTLARLASDFNLSNHIFKRCSKKLEEFEKTLLQAILENNSINRFQVQ
RYVSQSLVTLGSCSLLRKSIISLLLSEVNSFVDDLGAIDQDQGIYDVTRYENIFSLFWILEQVLQ
QAPQGDRTAHMDHSIPEMQTFLQKHDEVIFRLSDAFPLFAFYLWRLGVLLNSAEMETVKNESLP

>Arabidopsis_Prd2
MSSSVAEANHTEKEESLRLAIAVSLLRSKFHNHQSSSSTSRCYVSSESDALRWKQKAKERKKEII
RLQEDLKDAESSFHRDLFPANASCKCYFFDNLGVFSGRRIGEASESRFNDVLRRRFLRLARRRSR
RKLTRSSQRLQPSEPDYEEEAEHLRISIDFLLELSEADSNDSNFSNWSHQAVDFIFASLKKLISM
GRNLESVEESISFMITQLITRMCTPFKGNEVKQLETSVGFYVQHLIRKLGSEPFIGQRAIFAISQ
RISILAENLLFMDPFDESFPEMDECMFILIQLIEFLICDYLLPWAENEAFDNVMFEEWIASVVHA
RKAVKALEERNGLYLLYMDRVTGELAKRVGQITSFREVEPAILDKILAYQEIE
```

Discovered Motifs

1.

***E*-value:** 1.8e+001 **Site Count:** 2 **Width:** 6

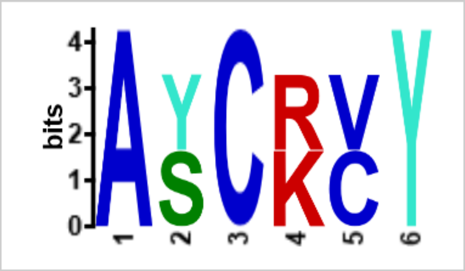

Log Likelihood Ratio: 36 **Information Content:** 22.9 **Relative Entropy:** 26.3 **Bayes Threshold:** 9.87344

Name	Start	*p*-value	Sites
5. Arabidopsis_Prd2	87	4.46e-9	SFHRDLFPAN ASCKCY FFDNLGVFSG
2. Yeast_Mei4	301	7.31e-9	HKLICINRKK AYCRVY LERELSLKKT

2.

***E*-value:** 1.8e+001 **Site Count:** 2 **Width:** 9

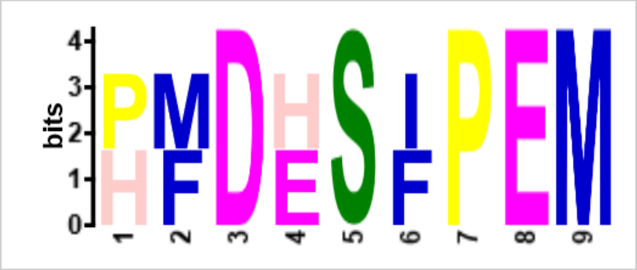

Log Likelihood Ratio: 52 **Information Content:** 34.9 **Relative Entropy:** 37.6 **Bayes Threshold:** 9.86186

Name	Start	*p*-value	Sites
5. Arabidopsis_Prd2	274	2.46e-12	ILAENLLFMD PFDESFPEM DECMFILIQL
4. Mus_MEI4	335	2.46e-12	QQAPQGDRTA HMDHSIPEM QTFLQKHDEV

3.

***E*-value:** 4.3e+001 **Site Count:** 2 **Width:** 9

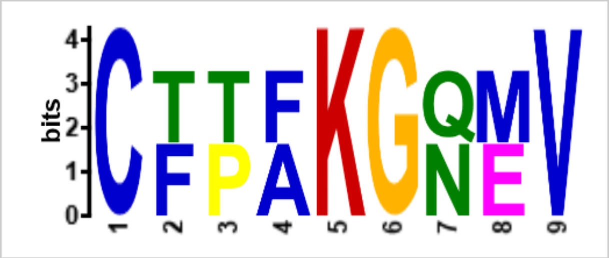

Log Likelihood Ratio: 51 **Information Content:** 33.9 **Relative Entropy:** 36.8 **Bayes Threshold:** 9.86186

Name	Start	*p*-value	Sites
5. Arabidopsis_Prd2	218	2.37e-12	FMITQLITRM CTPFKGNEV KQLETSVGFY
1. Tetra_Spop1	32	6.12e-12	AAALTSLQSN CFTAKGQMV SQNSQNSQNT

4.

***E*-value:** 1.3e+002 **Site Count:** 2 **Width:** 11

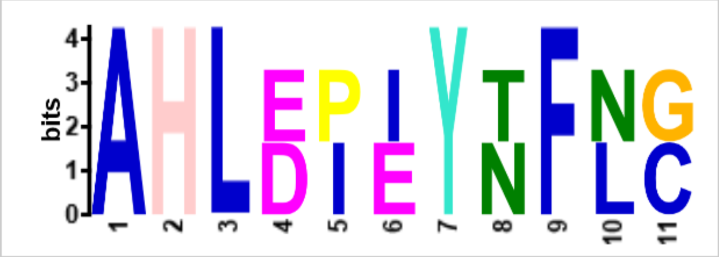

Log Likelihood Ratio: 59 **Information Content:** 41.5 **Relative Entropy:** 42.3 **Bayes Threshold:** 9.85409

Name	Start	*p*-value	Sites
3. pombe_rec24	129	8.82e-14	QEKRQTSGSL AHLDIEYTFNG LFRFLKCTAD
2. Yeast_Mei4	87	8.82e-14	NGFLNNKSLF AHLEPIYNFLC QNKYKSFEDA

5.

E-value: 1.6e+002 ? **Site Count:** 2 ? **Width:** 6 ?

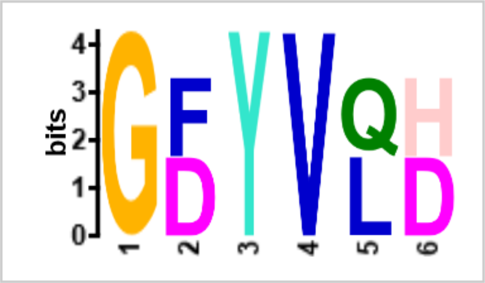

Log Likelihood Ratio: 34 ? **Information Content:** 22.9 ? **Relative Entropy:** 24.8 ? **Bayes Threshold:** 9.87344 ?

Name ?	Start ?	*p*-value ?	Sites ?
5. Arabidopsis_Prd2	234	1.37e-8	NEVKQLETSV GFYVQH LIRKLGSEPF
3. pombe_rec24	194	2.18e-8	PLVLTRLVEL GDYVLD LIIITQSIMQ

6.

E-value: 1.5e+002 ? **Site Count:** 2 ? **Width:** 6 ?

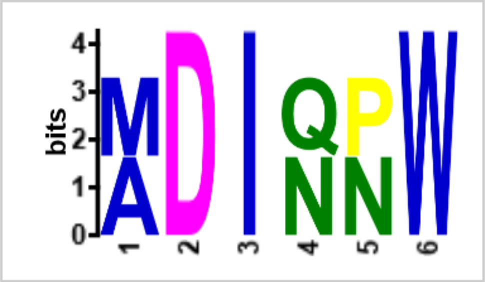

Log Likelihood Ratio: 36 ? **Information Content:** 22.9 ? **Relative Entropy:** 25.7 ? **Bayes Threshold:** 9.87344 ?

Name ?	Start ?	*p*-value ?	Sites ?
4. Mus_MEI4	1	1.98e-9	MDIQPW YLKTSKLALA
2. Yeast_Mei4	123	1.82e-8	LQSFSKDVST ADINNW FLPRYKILLK

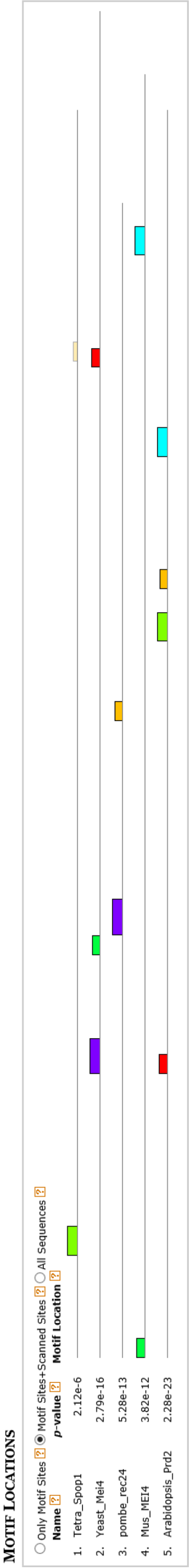

Figure S6

Structural features of *Tetrahymena* Pars11 and budding yeast RMM proteins and Hop1

Pars11

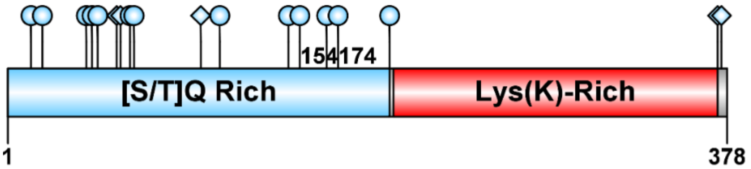

Putative phosphorylatable [S/T]Q sites were manually annotated.

Rec114

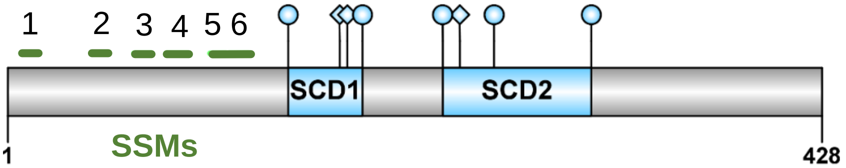

SCDs (S/T-Q cluster domains) are defined as regions where three or more SQ or TQ motifs are found within a tract of 100 residues or less (Traven and Heierhorst 2005, BioEssays 27: 397-407). SSMs, short signature sequence motifs (Tessé et al. 2017, Genes Dev. 31: 1880-1893). Phosphorylation sites (S/T-Q) annotated according to Carballo et al, 2013, PLoS Genet. 9, e1003545.

Mer2

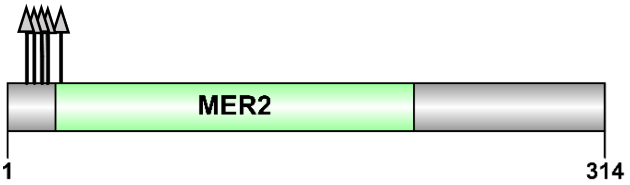

Cdc7-dependent Ser phosphorylation sites (Δ) according to Sasanuma et al. 2008, Nucl. Acids Res. 36: 984-997. Domain annotated according to the NCBI's Conserved Domain Database.

Mei4

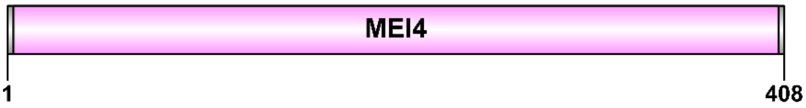

Domain annotated according to the NCBI's Conserved Domain Database.

Hop1

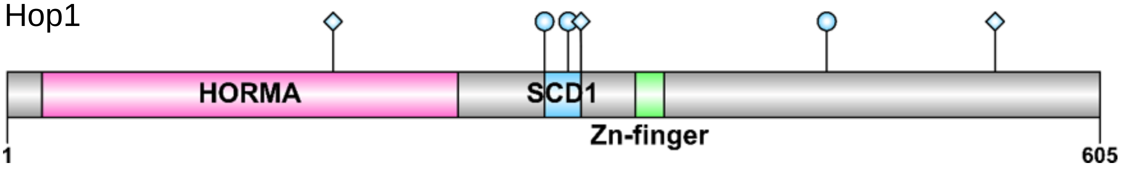

Phosphorylation sites (S/T-Q) were annotated according to Carballo et al. 2008, Cell 132: 758-770. The HORMA Domain was annotated according to the NCBI's Conserved Domain Database. The Zinc finger was annotated according to the Uniprot database. Domain annotated according to the NCBI's Conserved Domain Database.

Of the yeast RMM proteins, Rec114 most closely resembles Pars11 due to the presence of highly clustered S/T-Q motifs.No other similarities are found.

Diagrams were generated with the Illustrator for Biological Sequences (IBS) (Liu et al. 2015, Bioinformatics 31: 3359-3361).

Figure S7

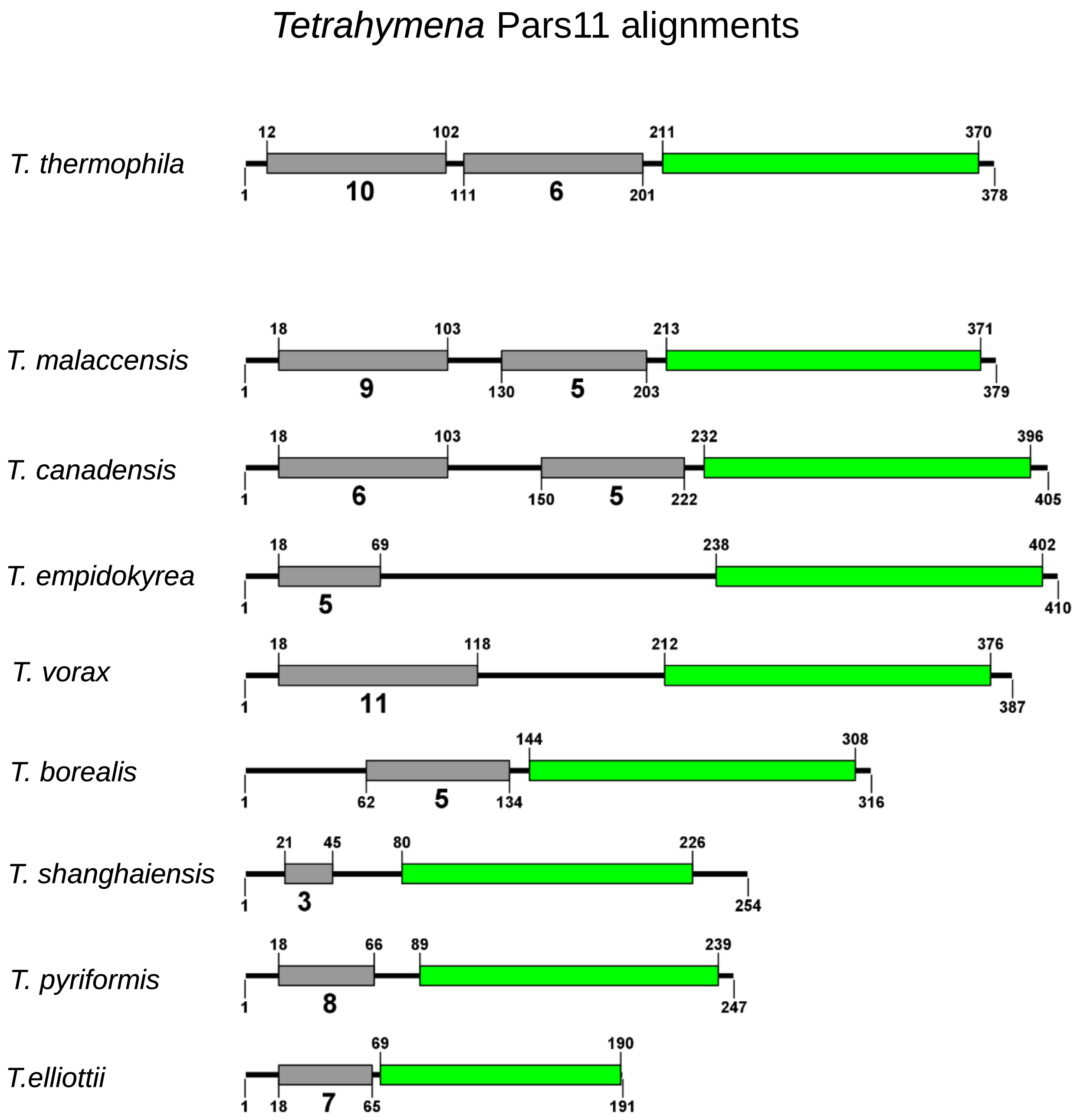

The S/TQ cluster domains are defined as regions where three or more SQ or TQ motifs are found within a tract of 100 residues or less. Gray bars indicate the N-terminal S/T-Q clusters, green bars the conserved Lys-rich C-terminus. Numbers under the S/T-Q clusters indicate the number of S/T-Q motifs. Diagrams were generated with the Illustrator for Biological sequences (Liu et al. 2015, Bioinformatics 31: 3359-3361).

Figure S7 continued

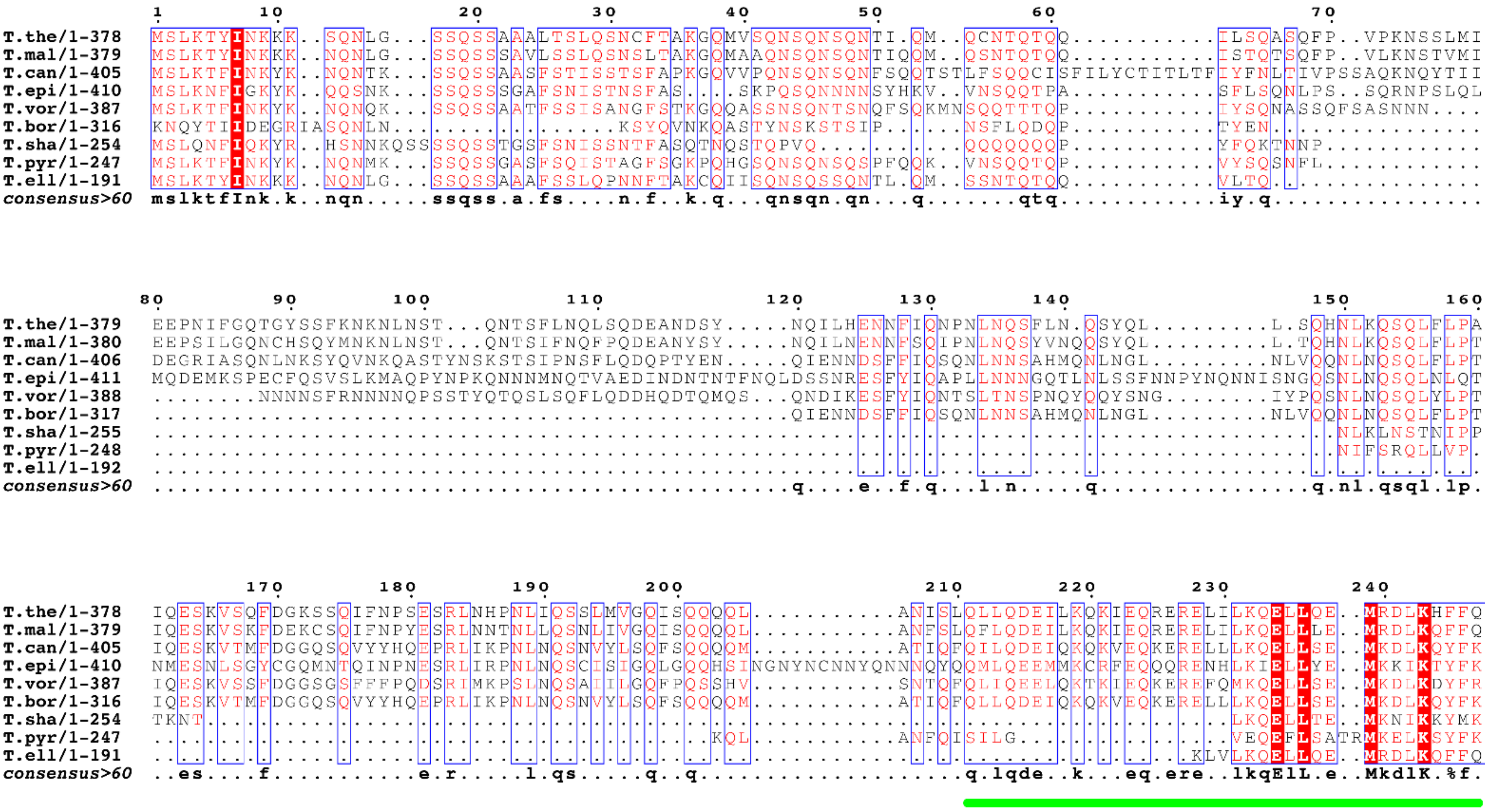

Figure S7 continued

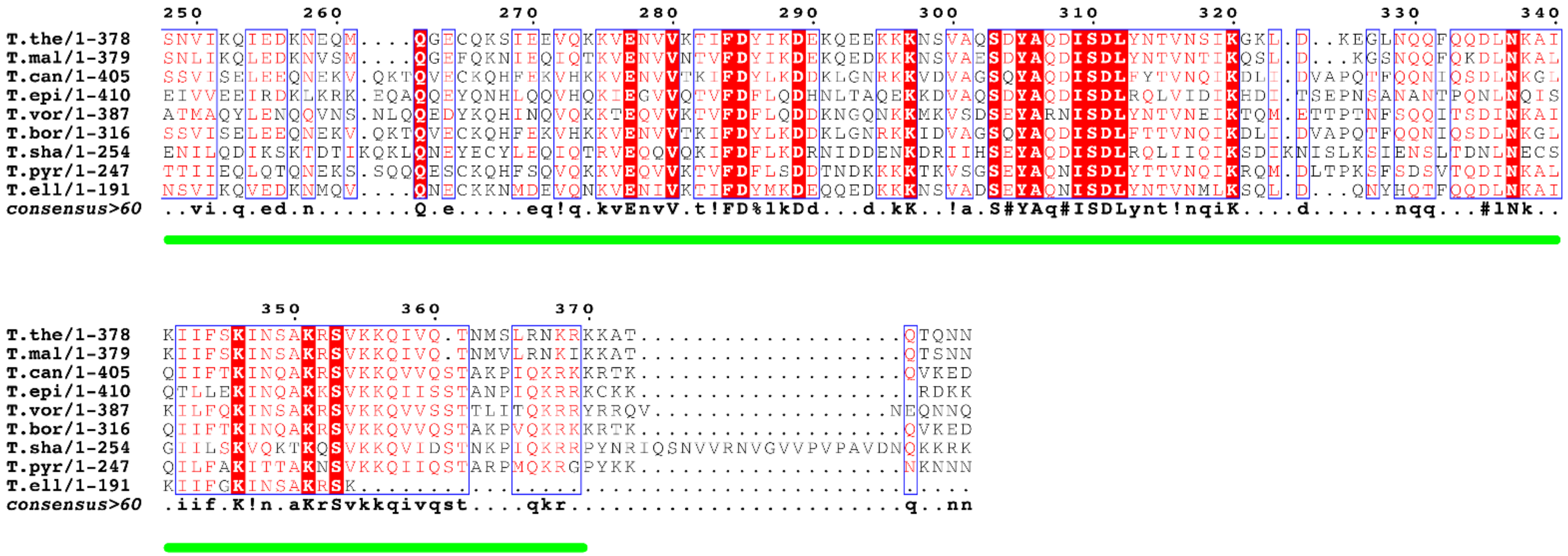

Orthologs of *T. thermophila* Pars11 proteins were identified by BLASTP or tBLASTn searches against proteomes or draft genomes of other *Tetrahymena* species in two databases (http://ciliate.org and http://ciliate.ihb.ac.cn). Protein sequences were aligned using MUSCLE (Edgar 2004, Nucl. Acids Res. 32: 1792-1797) and colored according to amino acid sequence similarity using Espript 3.0 (Robert and Gouet 2014, Nucl. Acids Res. 42 W1: W320-W324). Regions with >60% similarity are framed in blue. Identical residues are boxed in red, and similar residues are colored in red. The conserved C-terminal region was green underlined. The accession numbers of these proteins are: EIA_05075.1 (*Tetrahymena malaccensis*), TVORAX00043590 (*Tetrahymena vorax*), TSP00031430 (*Tetrahymena* canadensis), TEPIDO00188250 (*Tetrahymena* empidokyrea), TSHANG00212290 (*Tetrahymena shanghaiensis*), EI7_06775.1(*Tetrahymena elliotti*). Pars11 coding genes in *Tetrahymena borealis* and *Tetrahymena pyriformis* were incorrectly annotated, hence we corrected their annotation manually according to their genome sequences.
